# Sensitive Quantitative Proteomics of Human Hematopoietic Stem and Progenitor Cells by Data-independent Acquisition Mass Spectrometry*[Fn FN2]

**DOI:** 10.1074/mcp.TIR119.001431

**Published:** 2019-04-11

**Authors:** Sabine Amon, Fabienne Meier-Abt, Ludovic C. Gillet, Slavica Dimitrieva, Alexandre P. A. Theocharides, Markus G. Manz, Ruedi Aebersold

**Affiliations:** From the ‡Department of Biology, Institute of Molecular Systems Biology, ETH Zurich, 8093 Zurich, Switzerland;; §Hematology, University and University Hospital Zurich, 8091 Zurich, Switzerland;; ¶Functional Genomics Center Zurich, ETH Zurich and University of Zurich, 8057 Zurich, Switzerland;; ‖Faculty of Science, University of Zurich, 8057 Zurich, Switzerland

**Keywords:** Cell Sorting, Quantification, Mass Spectrometry, Differentiation*, Clinical Proteomics, Data-Independent Acquisition Mass Spectrometry, Fluorescence-Activated Cell Sorting, Hematopoietic Stem and Progenitor Cells, Low Cell Number, Proteomics

## Abstract

Physiological processes in multicellular organisms depend on the function and interactions of specialized cell types operating in context. Some of these cell types are rare and thus obtainable only in minute quantities. For example, tissue-specific stem and progenitor cells are numerically scarce, but functionally highly relevant, and fulfill critical roles in development, tissue maintenance, and disease. Whereas low numbers of cells are routinely analyzed by genomics and transcriptomics, corresponding proteomic analyses have so far not been possible due to methodological limitations. Here we describe a sensitive and robust quantitative technique based on data-independent acquisition mass spectrometry. We quantified the proteome of sets of 25,000 human hematopoietic stem/multipotent progenitor cells (HSC/MPP) and three committed progenitor cell subpopulations of the myeloid differentiation pathway (common myeloid progenitors, megakaryocyte-erythrocyte progenitors, and granulocyte-macrophage progenitors), isolated by fluorescence-activated cell sorting from five healthy donors. On average, 5,851 protein groups were identified per sample. A subset of 4,131 stringently filtered protein groups was quantitatively compared across the 20 samples, defining unique signatures for each subpopulation. A comparison of proteomic and transcriptomic profiles indicated HSC/MPP-specific divergent regulation of biochemical functions such as telomerase maintenance and quiescence-inducing enzymes, including isocitrate dehydrogenases. These are essential for maintaining stemness and were detected at proteome, but not transcriptome, level. The method is equally applicable to almost any rare cell type, including healthy and cancer stem cells or physiologically and pathologically infiltrating cell populations. It thus provides essential new information toward the detailed biochemical understanding of cell development and functionality in health and disease.

## Abstract (precis)

To date, technical limitations have precluded the robust quantitative proteomic analysis of rare cell types. We describe a highly sensitive mass spectrometry-based proteomic workflow for the analysis of human hematopoietic stem cells and three progenitor cell types. More than 5,000 protein groups could be consistently quantified from 25,000 sorted hematopoietic stem and progenitor cells. The data reproducibly identified characteristic patterns of differentially expressed proteins in the tested populations that indicated biochemical differences not apparent by transcriptomic analyses on equivalent samples.

## Graphical Abstract

**Figure:**
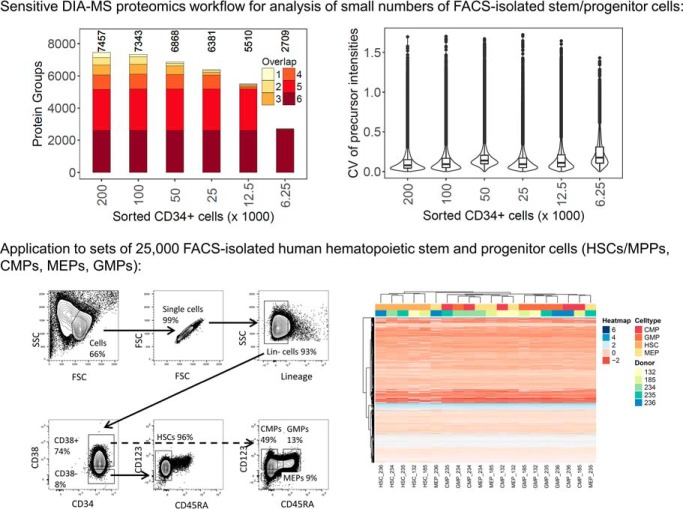

**Highlights**

- Sensitive DIA-MS proteomics workflow for analysis of small cell numbers.
- Application to healthy, FACS-isolated, human hematopoietic stem and progenitor cells.
- Good correlation between proteome and transcriptome for committed progenitors.
- Discrepant regulation of protein and mRNA in stem/multipotent progenitor cells.

In multicellular organisms, normal physiological functions and pathophysiological mechanisms are the result of the interplay of multiple cell types at various stages of differentiation. A prototypic example is the mammalian hematopoietic system where hematopoietic stem/multipotent progenitor cells (HSCs/MPPs) can differentiate into various functionally divergent cell lineages, including the downstream formation of common myeloid progenitors (CMPs)^1^, megakaryocyte-erythrocyte progenitors (MEPs), or granulocyte-macrophage progenitors (GMPs) (1, 2). When this process is altered, *e.g.* upon genetic or epigenetic changes in HSCs, abnormal, (pre)leukemic stem cell subpopulations may form, eventually resulting in clonal hematopoiesis and the onset of acute myeloid leukemia (3–5). To gain insight into the biochemical changes underlying cellular differentiation and to unravel factors involved in the early development of malignant hematopoietic diseases, highly refined analysis of the different cell subpopulations of the hematopoietic cell system is crucially needed (6).

Hematopoietic stem cells are critically rare compared with other hematopoietic cell types (7). Other numerically scarce, but functionally relevant, cell subpopulations include preleukemic stem cells (3–5, 8), leukemic stem cells (9), cancer stem cells in solid tumors (10, 11), circulating tumor cells (12, 13), and infiltrating T cells in solid tumors (14). Although the isolation of such rare cell types is supported by specific surface expression of cluster of differentiation (CD) markers such as CD34, CD38, CD123, CD45RA, and CD10 (15–17), normally no more than a few thousand cells per subpopulation can be isolated by fluorescence-activated cell sorting (FACS) from a single person. For example, the preparation of 25,000 sorted human HSCs requires up to 4 l of steady-state blood or a leukapheresis procedure following hematopoietic stem and progenitor cell (HSPC) mobilization, making further upscaling difficult. Whereas a few thousand cells can be routinely analyzed by modern imaging and genomic profiling technologies (1, 2, 16–19), proteome-level measurements, particularly the reproducible quantification of thousands of proteins across sample cohorts, has remained technically challenging for minute samples. Indeed, highly enriched human HSPC subpopulations have, to our knowledge, not been analyzed by unbiased large-scale proteomic analysis, even though global protein expression determines cellular functionality and provides critical information on the cellular differentiation process. Proteomic analysis of FACS-isolated cells has in general been reported only in studies focused on optimizing specific technical parts of the workflow, such as the cell sorting step itself (20), sample preparation (21, 22), or sample fractionation (23). Others used 400,000 cells as starting material, which restricted the scope of the analyses to large pools of murine samples (24) or *in vitro* model systems. Furthermore, no systematic assessment of the reproducibility or consistency of the proteomic results of small numbers of sorted cells has been performed, other than comparing protein identification numbers. It is therefore evident that the robust, reproducible, and quantitative proteomic analysis of minute samples, such as for example highly enriched HSPC, represents a significant technical and scientific advance.

Here, we present and apply an integrated workflow for the high-coverage, quantitative proteome profiling of minute amounts of sorted cells. It is based on data-independent acquisition (DIA)-MS on the Orbitrap Lumos platform and peptide centric signal extraction and analysis. DIA-MS is a massively parallel-in-time acquisition method of fragment ion mass spectra of all detectable precursors in a sample. It provides a complete, yet convoluted, quantitative fragment ion map record of a sample (25). Peptide-centric analysis (26, 27) of DIA datasets results in quantitative peptide matrices (25) of sufficient consistency and reproducibility to support label-free comparisons of large sample cohorts. To date, DIA studies on hybrid quadrupole-time-of-flight (QqTOF) (26, 28, 29) or Orbitrap (30, 31) platforms typically used microgram amounts of total peptide mass for analysis (and even larger amounts of actually processed starting material), a quantity that is one to two orders of magnitude above the quantity achievable by FACS isolation of rare hematopoietic cell types.

To overcome limitations of working with small amounts of proteins, we established a method to reproducibly identify and quantify nearly 6,000 protein groups with a median coefficient of variance (CV) of 9% for 125 ng of HEK293 tryptic peptides (see “Results”). This unprecedented performance was then used to profile minute amounts of highly enriched human HSCs/MPPs, CMPs, MEPs, and GMPs. The resulting protein *versus* sample data matrix revealed factors and biochemical pathways involved in quiescence, stemness maintenance, and cell differentiation. Comparison with RNAseq analyses demonstrated proteome-specific regulation of “stemness maintaining networks” in HSCs/MPPs.

## EXPERIMENTAL PROCEDURES

### 

#### 

##### Experimental Design and Statistical Rationale

Sample numbers: For method development, samples derived from cultured cells (HEK293 cell line) and human CD34+ hematopoietic stem/progenitor cells (isolated by FACS) were used in varying amounts as described. For final analysis of donor samples, material from five individuals (four cell types per donor) was analyzed, resulting in a total of 20 samples.

Replicates: HEK293 samples were analyzed in triplicates as technical replicates; CD34+ samples were processed in parallel as triplicates (process replicates). Due to the limited amount of sample material, donor samples were analyzed in single replicates, and different individuals were considered biological replicates. The number of samples/donors was limited by the material available from the hospital for this study.

Controls: Because this study did not involve a case/control design, no control samples were necessary.

Randomization: For LC-MS/MS analysis, the run order of donor samples (different individuals, different cell types) was randomized.

Statistical tests: Statistical procedures built into the software mapDIA (32) were used.

##### HEK293 Cell Culture

HEK293 cells (ATCC) were grown in Dulbecco's Modified Eagle's Medium (10% fetal bovine serum, 50 μg/μl penicillin, 50 μg/μl streptomycin) until confluence. Harvested cells were washed twice with 1x phosphate buffered saline and counted using an Invitrogen Countess automated cell counter (ThermoFisher Scientific, Waltham, MA). For the HEK293 peptide dilution series, 5 × 10^6^ cells were lyophilized and processed in bulk according to the protocol described below.

##### Human Hematopoietic Stem/Progenitor Cell Samples

Fresh human HSPCs were obtained from healthy stem cell donors (Clinical Hematology, University Hospital Zurich, Zurich, Switzerland) with informed consent and approval of the local ethics committee (KEK-ZH-Nr: 2015–0564). After mobilization with granulocyte-colony stimulating factor, stem cell-enriched mononuclear leukocytes were obtained via leukapheresis from peripheral blood for clinical stem cell transplantation. Any leftover material after clinical transplantation was collected for further cell preparation.

##### Cell Preparation, Flow-cytometric Analysis, and Cell Sorting

Human CD34+ hematopoietic stem/progenitor cells were enriched from mononuclear cells using immunomagnetic beads according to the manufacturer's instructions (CD34 MicroBead Kit; Miltenyi Biotec, Bergisch Gladbach, Germany). Following enrichment, CD34+ cells were frozen in liquid nitrogen. For analysis and sorting of hematopoietic stem cell-enriched cells and myeloid progenitors, CD34+ cells were thawed and stained with Tricolor/phycoerythrin (PE)-Cy5-conjugated antibodies specific for lineage markers: CD2, S5.5; CD3, 7D6; CD4, S3.5; CD7, CD7–6B7; CD8, 3B5; CD14, TuK4; CD19, SJ25-C1; CD56, MEM-188 (ThermoFisher Scientific-Invitrogen); CD10, HI10a; CD11b, ICRF44; CD20, 2H7 (BioLegend, San Diego, CA); CD235a, GA-R2 (BD Biosciences, Allschwil, Switzerland); and PE-Cy7-conjugated anti-CD34, 8G12 (BD Biosciences); fluorescein isothiocyanate-conjugated anti-CD38, HIT2 (BD Biosciences); allophycocyanin (APC)-conjugated anti-CD123, 6H6 (ThermoFisher Scientific-Invitrogen); and APC780-conjugated anti-CD45RA, HI100 (ThermoFisher Scientific-Invitrogen). Apoptotic cells were excluded in the analysis by Hoechst staining. Because less than 1% of all cells were apoptotic (see supplemental Fig. S1) and because of concerns regarding interference of the Hoechst stain with the mass spectrometric analysis, this step was omitted for further cell sorting. For compensation of the fluorochromes, cells and/or beads (anti-mouse Ig, kappa/negative control compensation particles; BD Biosciences) were singly stained with the antibodies. Compensation was performed automatically using the DIVA software (BD Biosciences) and checked manually.

For FACS analysis of intracellular isocitrate dehydrogenase 1 (IDH1) expression, the IntraPrep Leukocytic Permeabilization Reagent Kit (Beckman Coulter, Brea, CA) was used together with phycoerythrin (PE)-conjugated anti-IDH1, D2H1 (Cell Signaling Technology, Danvers, MA) and PE-conjugated isotype control, DA1E (Cell Signaling Technology).

All analyses were performed on a four laser-equipped LSR Fortessa machine (BD Biosciences), all sorting was performed on a five laser-equipped FACS Aria IIIu machine (BD Biosciences).

Gates were set using fluorescence minus one and unstained controls according to (17). The degree of purification of cell populations was determined by back-gating and double sorting (see supplemental Fig. S2) and determined to be >95% in all cases. FACS data were analyzed with FlowJo software (FlowJo LLC, Ashland, OR).

Highly enriched human HSCs/MPPs, CMPs, GMPs, and MEPs were isolated from five healthy HSPC donors (supplemental Table S1) using fluorescence-activated cell sorting and the cell surface markers CD34, CD38, CD123, and CD45RA. Lymphoid progenitors were excluded by adding the marker CD10 to the lineage mixture. For CD34+ cell dilution, purification control, fluorescence-minus-one, and validation experiments, CD34+ cells from an additional six healthy HSPC donors were used (ID 273, ID 227, ID 223, ID 183, ID 151, ID 340).

For MS analysis, 25,000 cells each were collected in 300 μl phosphate buffered saline solution in protein low-binding micro-centrifuge tubes (Eppendorf, Hamburg, Germany). All remaining material was sorted into separate micro-centrifuge tubes for the purpose of library generation. This resulted, depending on the donor sample and the cell type, in 13 additional samples (4x CMP, 4x HSC, 2x GMP, 3x MEP) with 40,000 to 270,000 cells. Samples were pelleted by centrifugation at standard force for viable HSPCs of 400 g for 15 min. We found that a critical step for reproducibility of material recovery was to very carefully remove the supernatant above the cells, leaving 50 μl on top of the pellet to avoid losing the nonadherent pelleted cells. The tubes were then snap frozen in liquid nitrogen, and the remaining FACS buffer was lyophilized.

##### Sample Preparation for Mass Spectrometry

The lyophilized cell pellets were resuspended in 10 μl (200 μl for bulk HEK293 preparation for the peptide dilution series) of 8 m urea in 100 mm ammonium hydrogen carbonate and lysed aided by sonication with a VialTweeter (Hielscher, Teltow, Germany) at an amplitude of 60%, a cycle of 60% and a duration of 20 s for three times with intermediate cooling on ice. One aliquot containing 128,000 HEK293 cells and one sample with 100,000 CD34+ hematopoietic stem/progenitor cells were submitted to a BCA protein assay following the manufacturer's guidelines (ThermoFisher Scientific-Pierce) to determine the protein content and the required amount of protease to be added.

Samples were diluted to 4 m urea with 100 mm ammonium hydrogen carbonate and treated with 1.25 U Benzonase Nuclease (Sigma-Aldrich, Darmstadt, Germany) per 25,000 cells for 30 min at 37 °C. Reduction of disulfide bonds was carried out by the addition of tris-(2-carboxyethyl)-phosphine (Sigma-Aldrich) to 5 mm and incubation at 37 °C for 30 min with shaking in a Thermomixer (Eppendorf), followed by alkylation of free thiol groups by the addition of iodoacetamide (Sigma-Aldrich) to 10 mm in the dark for 30 min at room temperature. Samples were diluted to 1 m urea, and sequencing grade trypsin (Promega, Madison, WI) was added at an enzyme-to-substrate ratio of 1:50 (extrapolated from the BCA assay results) for overnight digestion at 37 °C. After adjusting to 2% formic acid, samples were desalted with Empore Disks C18 (3 m, Saint Paul, MN) self-packed into StageTips format (33). Samples were dried using a vacuum centrifuge and resuspended in 11 μl of 2% acetonitrile and 0.1% formic acid with the addition of indexed Retention Time (iRT) peptides (34) (Biognosys, Schlieren, Switzerland) for the following MS analysis. An aliquot corresponding to 1,500,000 cells (285 μg protein) of the HEK293 sample was desalted with a 50-mg Sep-Pak tC18 cartridge (Waters, Milford, MA). After drying by vacuum centrifugation, the HEK293 sample was dissolved at 1 μg/μl in 2% acetonitrile and 0.1% formic acid with added iRT peptides. The peptide concentration was determined using the quantitative fluorimetric peptide assay (ThermoFisher Scientific-Pierce) following the manufacturer's instructions. The leftover material of the CD34+ samples after MS-injection was also submitted to peptide concentration determination.

For the generation of the HEK293 spectral library, an aliquot corresponding to 80 μg peptides was subjected to high pH reversed-phase peptide fractionation using a dedicated spin column kit (ThermoFisher Scientific-Pierce). Fractionation was carried out according to the manufacturer's recommendation. Briefly, eight fractions were collected by eluting with 300 μl each of increasing acetonitrile content (from 5% to 50% in 0.1% trimethylamine). The fractions were dried by vacuum centrifugation and redissolved in 25 μl of 2% acetonitrile, 0.1% formic acid with iRT peptides, of which 2 μl were injected per measurement. Additionally, unfractionated whole lysate of HEK293 was measured by LC-MS for the purpose of library generation.

##### Mass Spectrometry Analysis

Nanoflow LC-MS/MS measurements were carried out on an EASY-nLC 1200 system (ThermoFisher Scientific) coupled to an Orbitrap Fusion Lumos Tribrid mass spectrometer (ThermoFisher Scientific) equipped with a Nanospray Flex ion source.

Peptides were separated on an Acclaim PepMap 100 C_18_ column (ThermoFisher Scientific) with an inner diameter of 75 μm, a length of 25 cm, and a particle size of 2 μm. The column was operated at room temperature and at a flow rate of 300 nl/min. LC solvent A consisted of 98% water, 2% acetonitrile, and 0.1% formic acid, LC solvent B was composed of 80% acetonitrile, 20% water and 0.1% formic acid. Peptides were separated by a linear gradient from 5 to 37% B over 120 min, except for the purpose of library generation (240 min for unfractionated samples and 180 min for high pH reversed-phase fractions of HEK293, respectively).

The instrument was operated either in the DDA or DIA mode. In both cases, fragmentation was accomplished by higher energy collision dissociation at a normalized collision energy setting of 27%. The resolution of the Orbitrap analyzer was set to 120,000 and 30,000 for MS1 and MS2, with a maximum injection time of 100 ms and 50 ms, respectively. The mass range monitored in MS1 was 350–1,500 *m/z* and in MS2 200–1,800 *m/z* for DIA and the auto *m/z* normal scan range mode for DDA. The automatic gain control (AGC) target was set to 2e5 in MS1 and 5e5 or 8e4 in MS2 for DIA or DDA, respectively.

DDA measurements utilized the top speed setting, where one MS1 survey scan was followed by the acquisition of MS/MS spectra for a cycle time of up to a maximum of 3 s. In DIA mode, one MS1 scan was followed by 40 MS2 windows of equal width (15 *m/z*) with an overlap of 1 *m/*z, covering precursors in the range of 399.5–1,000.5 *m/z*. This resulted in a cycle time of 3.4 s. Data were acquired with Xcalibur 4.0.27.10 and Tune Plus version 2.1.

##### Data Analysis

DDA data were searched accordingly by Mascot (35) (Matrix Science, version 2.5.1) as well as Comet (36) version 2016.01 rev. 2 against the Swissprot reviewed subset of the human UniProt database (downloaded on 2016.07.06) containing 20,199 protein entries plus one additional protein entry for the concatenated iRT peptide sequences, plus as many appended decoy sequences generated by sequence reversal (keeping C-terminal K and R residues). The settings were as following: enzyme = trypsin, missed cleavages = ≤2, peptide tolerance = ±10 ppm, MS/MS tolerance = 0.02 Da, fixed modification = carbamidomethylation on cysteine, variable modification = oxidation on methionine.

Spectral libraries from DDA runs were generated as previously described (37). Briefly, the peptide-level false discovery rates (FDR) for the search results was independently adjusted to 1% for Mascot and Comet results using PeptideProphet (38) (TPP v4.7 rev 0 (39)). Mascot and Comet results were then combined by iProphet (40) and filtered to 1% protein FDR by Mayu (41) (v 1.08). Compilation of a consensus library used for the query of the DIA measurements was carried out by SpectraST (42, 43) (v 5.0) with the following filter criteria applied: include peptides at 1% peptide FDR for proteins at 1% protein FDR, six fragments per peptide, and fragment *m/z* range of 350–1,800 *m/z*. The HEK293 library included entries of 112,227 peptide precursors from 9,127 protein groups, the combined library for HEK293 cells and HSPC library samples 146,610 peptide precursors from 10,057 protein groups.

**Fig. 1.**
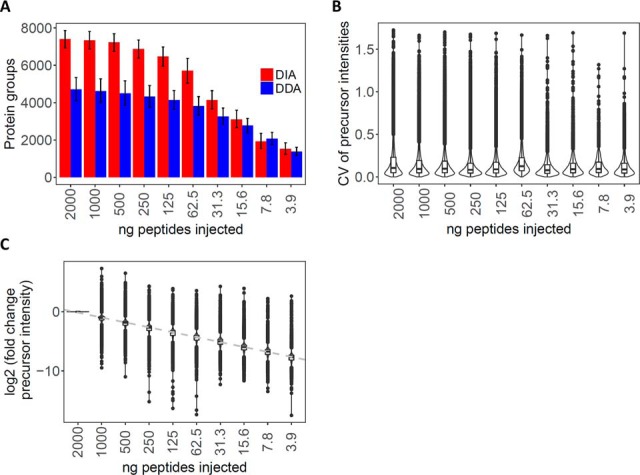
**HEK293 tryptic digests dilution series.** (*A*) Number of protein groups identified in DDA (blue) and DIA (red) mode, respectively, for decreasing loads of HEK293 tryptic peptides. The bars in the negative and positive directions represent the number of protein groups identified in common (intersection) or in total (union) for the technical triplicate injections at the indicated peptide loads, respectively. (*B*) Distribution of the CV for the peptide precursor intensities for the technical (process) triplicate injections for each sample load. (*C*) Distribution of the fold change (log2 scale) of the average precursor intensities between a given sample load and that at 2,000 ng sample load.

**Fig. 2.**
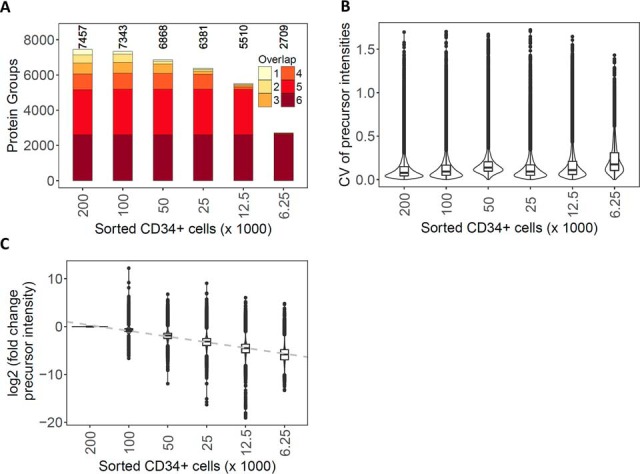
**Dilution series of human CD34+ hematopoietic cells isolated by FACS.** (*A*) Number of protein groups cumulatively identified across the technical replicates for decreasing numbers of FACS-isolated human CD34+ hematopoietic cells. The color scale represents the consistency of protein group identifications across the runs. (*B*) Distribution of the CV for the peptide precursor intensities for the technical (process) triplicate injections of processed FACS-isolated cells. (*C*) Distribution of the fold change (in log2 scale) of the average precursor intensities between a given sample load and that at 200,000 FACS-isolated human CD34+ hematopoietic cells.

DIA data were evaluated by Spectronaut 11 (31) (Biognosys) querying the above-mentioned library created from DDA runs with the following settings: data extraction with a tolerance of 10 ppm and 25 ppm for MS1 and MS2 level, with a dynamic retention time extraction window and automatic nonlinear iRT retention time calibration, identification at a precursor Q-value cutoff of 0.01 and protein Q-value of 0.01, quantification based on MS2 level area, and without cross-run normalization. The Spectronaut report (information on precursor, not fragment level) was exported for further processing in R. Protein groups were counted as defined by Spectronaut. For benchmarking datasets (HEK peptide dilution series and different collections of FACS-isolated CD34+ cell numbers), reports were used as such (without additional filtering, normalization or imputation) to generate the results for Fig. 1 and Fig. 2. CV calculations were computed in R on the per-triplicate dilution set level by dividing the standard deviation of the raw precursor intensities as reported by Spectronaut by the mean of the raw precursor intensities.

DDA data for the HEK293 dilution series were processed identical to the files for the library generation up to the step of adjusting the peptide FDR to 1% with PeptideProphet and compared with the corresponding DIA data at this level.

##### Proteomic Data Processing for HSPC Samples

The entries in the Spectronaut report were filtered to a protein Q-value cutoff of 0.01. We then filtered the data to keep only peptide precursors that were either (i) present in all donors for at least one cell type or (ii) in all cell types for at least one donor. Protein groups covered by only a single peptide were excluded from the differential analysis. The precursor quantitation matrix contained 785,280 entries, thereof 50,382 missing values (6.4%), for an average of 1.28 missing value per precursor across the 20 samples. Missing quantity values were imputed with random values in the range of 0.7 to 0.9 of the minimum value observed for the corresponding precursor. Normalization was performed based on the total ion current (normalization factor is calculated as mean of the sum of the precursor quantities across all measurements divided by sum of precursor quantities of the corresponding measurement).

The filtered, imputed, and normalized precursor data matrix was submitted to mapDIA v3.0.2 (32) for differential analysis. mapDIA calculates protein fold changes in a condition-based pairwise fashion by using the most stable peptides to perform the fold-change calculation. The replicate design set up was used, with standard deviation factor 2 and minimum correlation (median intraprotein correlation cutoff) 0.2.

For GO enrichment and search tool for recurring instances of neighboring genes (STRING) analysis, only unambiguous, single-protein entries from the results were considered and protein groups with multiple members ignored. This reduced the number of candidate proteins for these analysis steps to 3,364.

##### RNA Isolation, Quality Determination, and Sequencing

10,000 cells from each population were sorted into RNeasy lysis buffer containing beta-mercaptoethanol. Total RNA was purified according to manufacturer instructions using the RNeasy Plus Micro Kit (Qiagen, Hilden, Germany). RNA quality was determined using the Agilent 2100 Bioanalyzer System (Agilent Technologies, Santa Clara, CA). The samples demonstrated RNA integrity numbers ≥ 8.0 and were of high enough quality for a poly-A tail-based approach for RNA sequencing. RNA sequencing was performed as described in (44) using the Illumina HiSeq 4000 sequencing platform.

##### RNA Data Normalization and Processing

RNA sequencing data reads were quality-checked with FastQC (http://www.bioinformatics.babraham.ac.uk/projects/fastqc/). Reads were trimmed with Trimmomatic version 0.33 (4 and 3 bases were hard-trimmed from the start and end respectively; adapter trimming was done at the end). Trimmed reads were aligned to the Ensembl GRCh38 human reference genome and transcriptome with STAR version 2.5.1b (45). The average number of high-quality reads, reads aligned, and reads uniquely aligned per sample were 43.2 million, 42.6 million, and 35.9 million, respectively. Gene expression was quantified using the R/Bioconductor package Rsubread version 1.26.0 (46). Differentially expressed genes between cell types were identified using the R/Bioconductor package DESeq2 version 1.16.1 (47).

##### Gene Ontology Enrichment Analysis

For gene set enrichment analysis (GSEA), gene sets were retrieved from the Gene Ontology Consortium database (www.geneontology.org) on 2017–05-25 and 2017–05-27 (see supplemental Table S5). Ranked lists were created from the normalized and filtered proteome and transcriptome data using log2(fold change) or log2(fold change)x(-log10(adjusted *p* value)) as ranking criterion. GSEA was performed on the preranked lists using the GSEA software (v3.0, http://www.broadinstitute.org/gsea) with the minimum gene set size set to five and the remaining settings as defaults (19). Enrichments were deemed significant when FDR < 0.25 as suggested by (48). The number of genes used to calculate the enrichment scores depicted in Fig. 4*B* are shown in supplemental Table S6.

##### Network Analysis

Proteins significantly and differentially regulated compared with the corresponding mRNA were analyzed with STRING (http://www.string-db.org, v10.5) for their network connections. Significance was defined as FDR < 0.01 for both protein and mRNA data. Protein-protein interactions were computed based on the experimental and database evidence channels (49). The network was then loaded into Cytoscape using the STRING interaction score as distance and width for the edges. When available, the GO terms found enriched in Fig. 4*B* were mapped in color to the corresponding protein nodes.

##### Quantitative Polymerase Chain Reaction (qPCR)

Isolated mRNA (see above) was reverse transcribed according to manufacturer's instructions using the SuperScript IV VILO Master Mix with ezDNase enzyme (SuperScript IV Vilo Master Mix with ezDNase Enzyme, ThermoFisher Scientific). Quantitative analysis of cDNA was performed using Taqman probes and master mix (TaqMan Gene Expression Master Mix, ThermoFisher Scientific). Individual probes included Hs00271858_m1 (IDH1), Hs00255867_m1 (GAR1), Hs00950764_g1 (NHP2), and Hs99999903_m1 (ACTB) (ThermoFisher Scientific). ACTB was used as housekeeping control gene. Expression values were calculated using a Delta CT approach. Results were based on technical duplicates and biological triplicates.

## RESULTS

### 

#### 

##### Optimization of DIA-MS for Small Sample Loads

Most DIA-MS applications reported so far minimally used 0.5–2 μg of total peptide mass per injection (26, 28–31). To make the method compatible with the protein amounts extractable from FACS-isolated rare cells, we first optimized the acquisition scheme on an Orbitrap Fusion Lumos mass spectrometer to extend the application of DIA-MS to lower sample quantities, while minimizing attrition of quantified proteins and quantitative accuracy.

We developed and benchmarked the procedure with a peptide dilution series of HEK293 cells. Specifically, we refined sample handling as well as DIA-MS-specific measures (Fig. 1; see also supplemental text and Figs. S3-S9). In brief, the sample handling procedure minimized sample losses *e.g.* through surface adsorption, which are unavoidable when minute sample amounts are processed (supplemental Fig. S3). The mass spectrometric method maximized the use of ions injected into the mass spectrometer. Initially we monitored the ion density distribution in dependence of mass to charge (*m/z*) and retention time as a function of sample load (supplemental Figs. S4*A*–4*C*). We then optimized the DIA acquisition windows by adjusting the fill times. The benchmarking results obtained via the optimized MS method are summarized in Fig. 1. Fig. 1*A* shows a comparison of the number of protein groups identified by standard DDA and the optimized DIA method as a function of sample load. The results indicate that for sample loads above 30 ng the DIA mode consistently identified a higher number of peptide precursors and protein groups than DDA. Fig. 1*B* shows an assessment of the quantitative reproducibility and accuracy of data generated in DIA mode. The measurements achieved an average peptide quantification CV of less than 10% for triplicate injections across the whole dilution series. Further, the peptide quantification values obtained for the consecutive dilution steps retained linearity throughout the entire dilution range (Fig. 1*C*). We also show that, at 30 ng sample loads, longer fill time increases further the number of identifications (supplemental Figs. S4*D*–S4*F*), demonstrating that DIA performance is optimal at very low sample loads by leveraging the fill time capabilities of ion-trap instruments. In summary, the results indicate that an optimized economy of the available precursor ions on the Orbitrap Lumos significantly extends the performance of DIA proteomics toward sample amounts in the low nanogram range with minimum attrition in terms of identified proteins and quantitative accuracy.

To assess the reproducibility and accuracy of the peptide/protein identification and quantification for the whole procedure, we collected from the FACS instrument triplicate samples of 200,000; 100,000; 50,000; 25,000; 12,500; and 6,250 human CD34+ hematopoietic stem/progenitor cells and processed them with our sample preparation procedure in parallel. We determined that ca. 3.2 μg of total peptide mass could be recovered from 200,000 sorted cells after the entire process, of which ∼73% (8/11 μl) could be injected by the autosampler for DIA measurements. By extrapolation, we therefore estimated that we injected ∼2,327; 1,164; 582; 291; 145; and 73 ng of peptides, respectively, from the lower numbers of sorted cells. In comparison, 26.8 μg of peptide mass was obtained from 200,000 HEK293 cells when processed in bulk. This difference in the amount of peptides obtained from the same number of HEK293 and hematopoietic stem/progenitor cells is roughly in agreement with the 4–5 times smaller cell volume expected for the human CD34+ hematopoietic cells compared with HEK293 cells (50, 51) and the expected increase in loss from processing low sample amounts.

Along the dilution series of FACS-isolated cells the average number of identified protein groups decreased from 6,955 for 200,000 to 4,833 for 12,500 sorted human CD34+ hematopoietic cells (Fig. 2*A*). A median CV below 14% (Fig. 2*B*, supplemental Table S2) and a good linearity of quantification was maintained for these measurements (Fig. 2C). At 6,250 cells, the number of identified protein groups decreased to 2,248 and the CV of quantification increased to above 17%. However, the overall peptide quantification remained well correlated throughout the entire CD34+ series (supplemental Fig. S9), indicating that protein quantification remained quite accurate even for samples containing as few as 6,250 cells. The 2,709 protein groups identified in these samples were almost entirely subsumed in the set of proteins identified from higher cell numbers (supplemental Fig. S10), indicating that after normalization of signal intensities, meaningful comparisons across sample cohorts are feasible, even in cases in which one or several samples are only available in minute quantities.

Relating the results from sorted human CD34+ hematopoietic cells to those of the HEK293 peptide dilution series, we noted an attrition in the number of identified proteins and their quantification accuracy for cell numbers below 25,000, corresponding to <300 ng of peptide mass on column. Overall, the optimized DIA and sample preparation method provided reproducible identification and quantification results (in all three replicates) for more than 5,100 protein groups from as little as 25,000 FACS-isolated human CD34+ hematopoietic cells.

##### Proteomic Analysis Underscores Ontogenetic Distance Between Individual Human Hematopoietic Cell Types

**Fig. 3.**
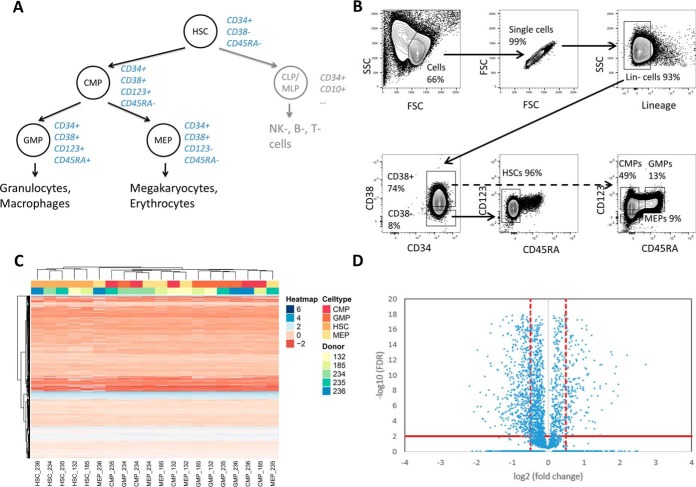
**Proteome profiles of human hematopoietic stem and progenitor cell subpopulations.** (*A*) Human hematopoietic cell hierarchy with respective cell surface markers depicted in blue (15–17). (B) FACS strategy, depicted on magnetic-activated cell sorting-preselected CD34+ hematopoietic cells isolated from healthy HSPC donors. Shown are the analysis gates. Highly enriched HSCs/MPPs (referred to as HSCs) are CD34+CD38-CD45RA-, highly enriched CMPs are CD34+CD38+CD123+CD45RA-, highly enriched GMPs are CD34+CD38+CD123+CD45RA+, and highly enriched MEPs are CD34+CD38+CD123-CD45RA-. (*C*) Nonsupervised hierarchical clustering (Euclidean distance) heatmap (78) of intensities for the peptides identified in HSCs, CMPs, GMPs, and MEPs (shades of red) isolated from five different donors (shades of blue). The peptide intensities are centered and scaled and depicted in color shades from red to blue. The missing peptide intensity values are shown in white. (*D*) Volcano plot of differential analysis of proteins. Comparison of HSCs to the average of the three other cell types. Abbreviations: HSPC, hematopoietic stem and progenitor cell; HSC, hematopoietic stem/multipotent progenitor cell; CMP, common myeloid progenitor; CLP/MLP, common/multipotent lymphoid progenitor; GMP, granulocyte-macrophage progenitor; MEP, megakaryocyte-erythrocyte progenitor; SSC, side scatter; FSC, forward scatter.

We applied the newly developed method to profile the proteome of human CD34+ hematopoietic cell subpopulations isolated from the peripheral blood of five HSPC donors (age 28–57 y, see supplemental Table S1). Four highly enriched subpopulations, including rare HSCs/MPPs, CMPs, MEPs, and GMPs, respectively, were isolated by FACS (Figs. 3*A* and 3*B*), processed and analyzed by DIA-MS. Guided by the cell dilution experiment described above, 25,000 cells were collected for each subpopulation. To support peptide-centric analysis of the DIA data, we generated a spectral library specific for the cell types of this study (see “Experimental Procedures”). We identified on average 5,851 protein groups for the different human HSPC populations (supplemental Fig. S11, supplemental Table S3). To increase the robustness of the following differential comparison, we applied additional stringent filtering criteria that resulted in a final list of 4,131 protein groups (from 39,264 peptide precursors) that were quantified consistently with at least two peptides across the samples.

Because the cell samples were derived from nonrelated donors of different age and the analyzed cell populations are relatively close in the cell differentiation tree, we first tested whether the quantitative protein measurements were sufficiently accurate and reproducible (see supplemental Figs. S12 and S13) to confidently detect cell subtype-specific differences despite the expected interperson variability. The summary heatmap of these comparisons (Fig. 3*C*) indicates that the proteome profiles clustered according to cell subtype rather than donor. HSCs/MPPs clustered the furthest away from the other cell subpopulations, while CMPs were found to be more similar to GMPs and MEPs for some donors, in agreement with the ontogenetic distances expected between the different cell lineages (Fig. 3*A*).

Differentially expressed proteins were detected in the various cell subpopulations with roughly similar numbers of proteins being significantly up- or down-regulated, whereby significance was defined by the cutoffs of FDR <0.01 and log2(fold change) >0.5 (Fig. 3*D* and supplemental Fig. S14). Cutoffs were chosen based on the protein fold-changes observed in the volcano plots (supplemental Fig. S14*A*). Using these cutoffs and comparing HSCs/MPPs to the average of all three remaining subpopulations (CMPs, GMPs, MEPs) (52), 1,008 proteins were determined to be differentially abundant in HSCs/MPPs. In accordance with the close ontogenetic distance of CMPs to GMPs and MEPs, the number of proteins with significantly changed expression between these cell types was somewhat lower: for GMPs 489, for MEPs 370, and for CMPs 64.

The availability of DIA data for each cell type from five donors allowed us to confidently identify proteins that were consistently detectable in some cell types but expressed below the detection limit of the measurement in other cell types and thus provided particularly important biological information (see supplemental Fig. S15). For example, the enzyme myeloperoxidase was consistently detectable at a low level in CMPs and at a much higher level in GMPs but was below the limit of detection in MEPs and HSCs/MPPs. The proliferation marker protein Ki-67 was not detected in HSCs/MPPs but was consistently detected in CMPs, GMPs, and MEPs. Similarly, all five members of the condensin-1 complex (SMC2, SMC4, NCAPD2, NCAPG, and NCAPH), a complex responsible for chromatin condensation, were present below the detection limit in HSCs/MPPs but could be clearly detected in the other cell types studied.

Overall, these results show the consistent detection of quantitative protein patterns that are characteristic for ontogenetically close cell types.

##### Gene Ontology Enrichment Analyses for Proteomics and Transcriptomics Data

**Fig. 4.**
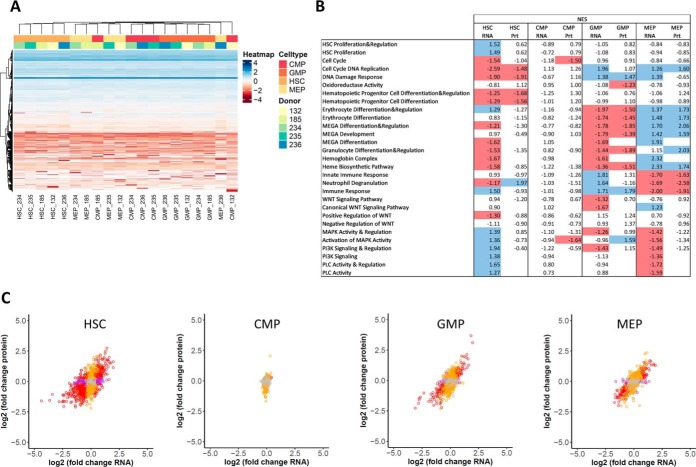

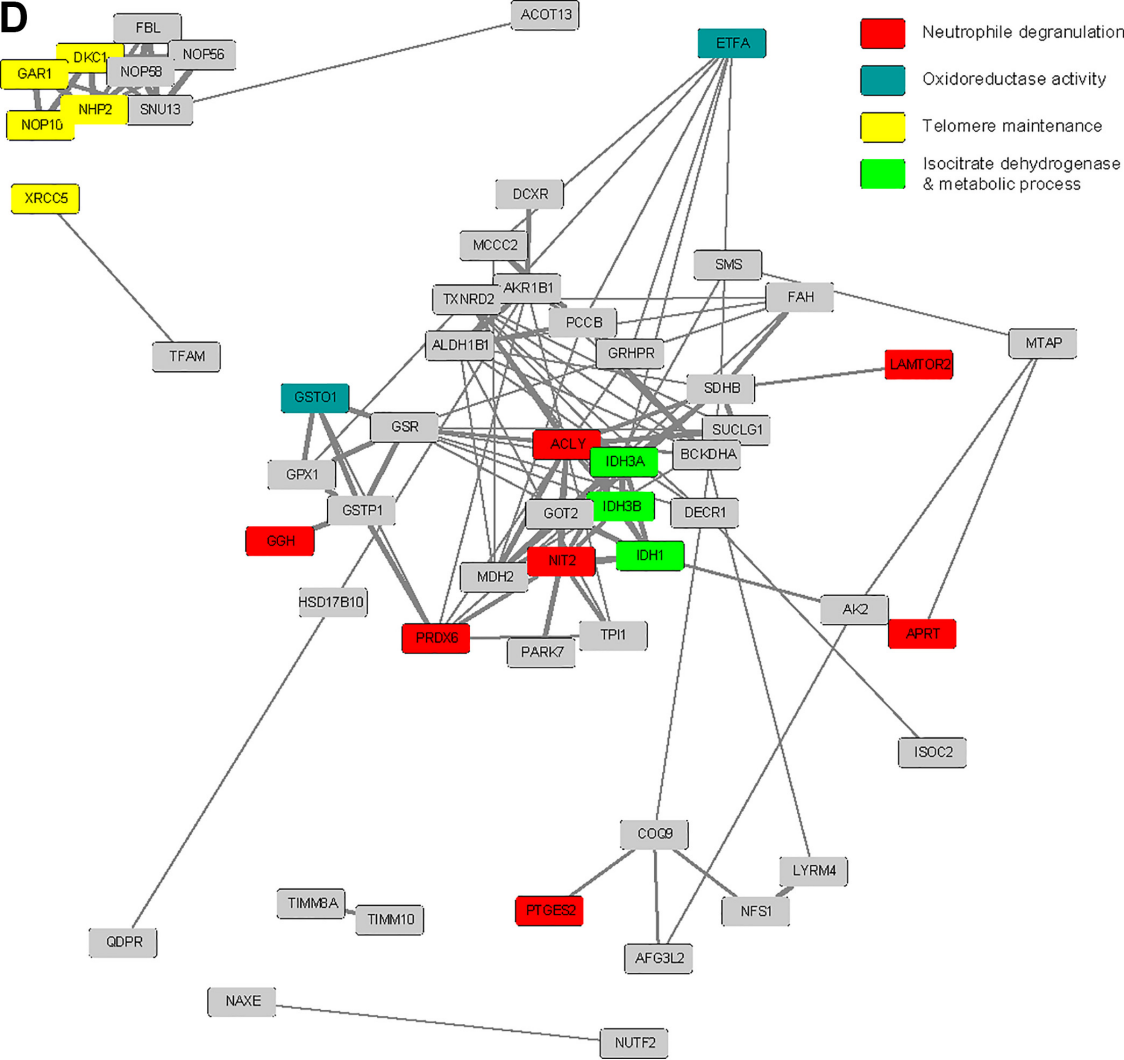
**Proteome-transcriptome correlation in human hematopoietic stem and progenitor cell subpopulations.** (*A*) Nonsupervised hierarchical clustering (Euclidean distance) heatmap (78) of the intensity for the transcripts identified in HSCs/MPPs (referred to as HSCs), CMPs, GMPs, and MEPs (shades of red) isolated from five different HSPC donors (shades of blue). The transcript intensities are centered and scaled and depicted in color shades from red to blue. The transcripts with missing transcript intensity values in all samples were removed because they could not be handled by the clustering algorithm. Remaining missing transcript intensity values are shown in white. Clustering was observed mostly according to cell type, not according to donor. (*B*) GO enrichment analysis showed good alignment of protein and mRNA data. GSEA was performed for ranked mRNA and protein lists using GO processes from the Gene Ontology Consortium database as gene sets. Shown are normalized enrichment scores for the individual gene sets. Significantly up-regulated gene sets are marked in blue color; significantly downregulated gene sets are marked in red color. Significance was defined as FDR < 0.25, specific cell subpopulations were compared with the average of the remaining three cell types, and log2(fold change) was used as ranking criterion. Empty fields mean that no enrichment could be calculated. Abbreviations: MEGA, megakaryocyte; MAPK, mitogen-activated protein kinase; PI3K, phosphoinositide-3-kinase; PLC, phospholipase C. (*C*) Correlation between proteomics and transcriptomics data for HSCs/MPPs (referred to as HSCs), CMPs, GMPs, and MEPs. Dots are depicted in red when the FDR was below 0.01 both for protein and RNA data, orange when FDR < 0.01 for protein data, purple when FDR < 0.01 for RNA data, and gray when FDR ≥ 0.01 for both protein and RNA data. (*D*) Network analysis of significantly up-regulated proteins with concomitant significantly downregulated mRNA in HSCs/MPPs. Two clusters were especially prominent, including the snoRNPs and telomerase complex proteins GAR1, DKC1, NOP10, NHP2, and the quiescence-inducing NAD(P)H-producing proteins IDH1, IDH3A, and IDH3B. HSCs/MPPs were compared with the average of the other three subpopulations; cutoffs were set at FDR < 0.01 for protein and RNA data. Colors depict GO terms found enriched in Fig 4*B*.

From the same sorting experiments, a further 10,000 cells were isolated for RNAseq. Similar to the proteomic results, the transcriptomic data also revealed clustering mostly by cell type, rather than by donor (Fig. 4*A*). As for proteins, the HSCs/MPPs transcript profiles clustered furthest apart from the other cell subpopulations, whereas CMPs were more similar to GMPs and MEPs.

To further assess the proteomic and transcriptomic results, we performed gene set enrichment analyses for specific GO processes involved in hematopoietic stem cell differentiation according to previous studies and functional annotations (1, 2, 18, 19, 24, 48, 52, 53). The proteomic and transcriptomic data closely recapitulated most of the expected changes in GO processes (Fig. 4*B*, supplemental Figs. S16 and S17). Cell cycle/DNA replication/DNA damage response was found downregulated for HSCs/MPPs, which are more quiescent than progenitor cells, both at the protein and mRNA level. Erythrocyte differentiation/megakaryocyte development/heme biosynthesis were observed to be up-regulated in MEPs at the protein and mRNA level. (Innate) immune responses were found up-regulated in GMPs at the protein and mRNA level. The canonical WNT pathway was observed to be up-regulated in MEPs at the mRNA level. Mitogen-activated protein kinase, phosphoinositide-3-kinase and phospholipase C pathways were all shown to be up-regulated in HSCs at the mRNA level (1, 2, 18, 19, 52, 53). Transcription factors Gata1 and Gata2 were also manually validated to be present in the CMP and MEP cell types only (supplemental Fig. S15), as expected.

Of note, the proteomic and transcriptomic results showed highest agreement of the GO enrichments for the GMPs and MEPs, both in terms of directionality (up or down) and significance of the pathway enrichments. For the CMPs, only a few significantly enriched pathways were observed, probably due to the position of CMPs between GMPs and MEPs in the developmental system. Interestingly, the HSCs/MPPs showed fewer significantly up- or downregulated GO processes for the proteomics data compared with the transcriptomics results (*e.g.* HSC proliferation, mitogen-activated protein kinase (MAPK) activity and regulation, phosphoinositide-3-kinase (PI3K) signaling, phospholipase C (PLC) activity (Fig. 4*B*)). This was in part due to the higher number of transcripts observed (17,355) compared with the number of detected protein groups (4,131).

Overall, the GO pathway analysis was in agreement with the expected properties of the respective cell types, thereby validating our protein and mRNA results against former studies.

##### Discrepant Protein and mRNA Regulation in Highly Enriched HSCs/MPPs

To investigate the complementary value of the protein quantitative information, we decided to examine proteins that were found differentially regulated compared with their mRNAs (Fig. 4*C* and supplemental Figs. S14*B*–14*E*).

The protein *versus* mRNA fold-change plots (Fig. 4*C*) showed good correlation for MEPs (R^2^ of 0.50) and GMPs (R^2^ of 0.41) but somewhat lower values for the HSCs/MPPs (R^2^ of 0.32) and CMPs (R^2^ of 0.06), in line with the results from GO enrichment analysis (Fig. 4*B*).

STRING analysis of proteins differentially regulated at the protein and transcript level revealed two major protein-protein association networks in HSCs/MPPs, the first including the small nucleolar ribonucleoproteins (snoRNPs) and telomerase maintenance proteins GAR1, DKC1, NOP10, and NHP2 and the second, the quiescence-inducing NAD(P)H-producing isocitrate dehydrogenase proteins IDH1, IDH3A, and IDH3B (Fig. 4*D*, Experimental Procedures). Both association networks were up-regulated on protein (see also supplemental Figs. S15 and S18*B*) and downregulated on mRNA level in HSCs/MPPs (see supplemental Fig. S18*A*).

## DISCUSSION

HSCs are mostly quiescent cells, *i.e.* a major fraction of cells is expected to be in G0 of the cell cycle. These cells have been shown to contain very low levels of mRNA, while still maintaining a relatively constant protein mass overall. Proteins may therefore provide a better readout of the cellular state of quiescent cells than the mRNAs. The same may be postulated for relatively quiescent (pre)leukemic and cancer stem cells. Until now, extensive global proteome data could not be obtained in critically rare cell types such as human HSPC subpopulations or (pre)leukemic and cancer stem cells. To achieve the most comprehensive and accurate protein quantification presently possible for minute, clinically relevant samples, we developed and applied an integrated sample preparation/DIA-MS method, using an Orbitrap instrument. We report several critical observations for sample preparation to avoid loss of cell pellets and of hydrophobic peptides (supplemental Fig. S3) and regarding optimization of ion trap fill times in DIA (supplemental Fig. S4).

We applied our newly developed DIA acquisition scheme to the analysis of 25,000 FACS-isolated human HSPC subpopulations and could quantify more than 5,800 protein groups per cell subtype. Of these, we used a stringently filtered subset (4,131 protein groups) for further analysis. Importantly, our results also demonstrate that even though the number of protein identifications decreased when lowering the number of cells further to 12,500 or even 6,250 cells, quantification retained a very high level of accuracy (Figs. 1*C* and 2*C*, supplemental Figs. S7 and S9). In practice, this means that, even if fewer than 25,000 cells are available from FACS, protein quantification results can still be confidently compared with those from higher cell counts, if the proteins are detectable in both samples.

Analysis of key marker proteins with strong differences in expression between individual HSPC subpopulations demonstrated the expected results for the proteome data. In line with its role in neutrophil immune reactions and its localization in the alfa granules of granulocytes (54), the enzyme myeloperoxidase was not detected in HSCs or MEPs, seen at low protein levels in CMPs and at high levels in GMPs. Myeloperoxidase is a marker for granulocytes, and its presence can be used to distinguish between myeloid and lymphatic origins of acute leukemias (54). The presence of myeloperoxidase mainly in GMPs is thus in agreement with the differentiation potential of GMPs to granulocytes (55). Furthermore, in line with the quiescent state of HSCs/MPPs, proliferation and mitotic markers such as Ki-67 and condensin-1 complex members were not detected in this cell type (56, 57). These results provided validation of the proteome data in HSPC samples with critically low cell numbers, thereby allowing for comparisons of proteome and respective transcriptome data.

Proteome and corresponding transcriptome data showed the same clustering pattern. Moreover, cell subpopulations were more decisive for the clustering than genomic variability, indicating that the data quality achieved allowed us to detect biologically relevant protein patterns in a noisy background. Furthermore, enrichment analyses for GO terms in the different HSPC subpopulations reinforced the good alignment of protein and transcript data, thereby further validating the quality and information content of the proteomic results. Whereas GMPs and MEPs had very similar GO enrichment results for proteins and transcripts, HSCs/MPPs—though overall well aligned for proteomics and transcriptomics—showed fewer significantly up- or downregulated GO processes for the proteomics data, compared with the transcriptomics results (*e.g.* HSC proliferation, MAPK activity and regulation, PI3K signaling, PLC activity). This is in part due to the lower coverage achieved for proteins compared with transcripts. In addition, this could also indicate the presence of alternative processes regulating the abundance of transcripts and proteins (58). Given the similar data quality (number of protein groups identified and quantified, CV) for the various analyzed cell types, the different behavior of HSCs/MPPs is likely to be biologically significant and may at least in part reflect the noncycling state of HSCs/MPPs as opposed to the cycling state of hematopoietic progenitor cells.

In line with these findings, proteins that showed discrepant regulation between their proteomic and transcriptomic data (59) were observed almost exclusively in HSCs/MPPs. Buffering of mRNA alterations at the level of protein concentrations is a well-known phenomenon, illustrating that transcript levels by themselves are not sufficient to predict protein levels in many scenarios (60). In HSCs/MPPs two clusters were identified, which are up-regulated on the protein level while downregulated on the mRNA level in HSCs/MPPs. The first is a strongly interconnected protein module that includes several snoRNPs and telomerase maintenance proteins and is deemed essential for long-lived stem cells (61–63). Telomerase activity in hematopoietic cells is associated with self-renewal potential and has been shown to decrease upon myeloid differentiation (62). Mutations in these telomerase maintenance proteins result in dyskeratosis congenita, a syndrome characterized by bone marrow failure and an increased risk for acute myeloid leukemia and myelodysplastic syndromes (64). Differential protein and mRNA regulation has been reported for these proteins and was attributed to posttranslational mechanisms (63, 65). The second cluster consisted of the IDH proteins IDH1, IDH3A, and IDH3B that have previously been shown to maintain quiescence in hair follicle stem cells (66). IDHs are also thought to play a key role in hematopoietic stem cell homeostasis and were reported to be mutated in ∼20% of acute myeloid leukemias (67, 68). IDH proteins catalyze the oxidative decarboxylation of isocitrate to alpha-ketoglutarate and are involved in adaptation to hypoxia, histone demethylation, and DNA modification (69). IDH1 is a cytosolic/peroxisomal homodimer whereas IDH3 is a mitochondrial heterotetramer composed of two alpha, one beta, and one gamma subunits (70–72). Mutant IDH enzymes have neomorphic activity, leading to the formation of the (R) enantiomer of 2-hydroxyglutarate and causing DNA and histone hypermethylation, altered gene expression, and blocked differentiation of hematopoietic progenitor cells (69). Acute myeloid leukemia treatments targeted at mutant IDH proteins have entered clinical routine, such as the IDH2 inhibitor enasidenib that received FDA approval for the treatment of relapsed or refractory acute myeloid leukemia on August 1, 2017 (www.fda.gov). Other IDH inhibitors are currently being evaluated in clinical trials (69).

qPCR validation experiments supported down-regulation of these targets in HSCs relative to the other HSPC subpopulations, whereas flow cytometry validation experiments confirmed high IDH1 protein expression in HSCs (supplemental Figs. S18*A*, S18*B*, and S19). In contrast to the proteomics data, equally high IDH1 flow cytometry intensity was seen in HSCs relative to CMPs, which could be due to differences in subcellular distribution of IDH1 in for example peroxisomes (71, 72).

These examples illustrate the relevance of generating high-quality proteomic data for well-defined cell subpopulations for the identification of biological processes that cannot be detected by genomic or transcriptomic analysis. Though this seems particularly evident for quiescent cells, we expect that proteomic data will bring an invaluable layer of biological information complementary to that of the transcriptomic data for many other cell subtypes. The presented application of DIA to acquire robust protein quantification data on low sample loads can thus be expected to increase our understanding of the dynamics of cell type-specific networks and to complete our knowledge on differentiation processes at play in healthy and pathological numerically scarce cells (19, 52).

In future research, it will be important to further refine the different cell subpopulations. CD34+CD38-CD45RA- HSCs/MPPs, CD34+CD38+CD123+CD45RA- CMPs, CD34+CD38+CD123+CD45RA+ GMPs, and CD34+CD38+CD123-CD45RA- MEPs could indeed be further divided into biologically even more refined subpopulations (2, 16). Also, proteomic data from cell types isolated directly from bone marrow or from cord blood rather than from mobilized HSPCs obtained from donors after artificial stimulation will need to be obtained. Those new samples will enable to increase the number of peptide assays in the spectral library, potentially allowing to identify additional proteins in the present and future DIA datasets. Further fine tuning of the sample handling steps may be necessary to cope with possibly even lower sample amounts than those analyzed in this study. The dilution series in HEK cells demonstrated a faster than linear signal drop-off for hydrophobic peptides suggesting adsorptive losses to surfaces. Possible avenues to explore would be to miniaturize cell lysis and digestion with the help of novel microfluidic devices (73) and to use LC columns with further reduced inner diameters (74). Thus, by combining improvements in sample processing and instrument design, it can be expected that the method will allow to robustly quantify proteins from even lower sample loads. Furthermore, transcription factors are of low abundance and are therefore difficult to detect, especially by large-scale proteomic analysis without fractionation. Specifically concerning the Hox and Gata genes, we checked our spectral library and confirmed that it contained peptide assays for 12 HOX proteins and 4 GATA proteins, almost all exclusively originating from the human HEK fractionation library. Future developments in DIA-MS analysis are required for reliable detection of such key low abundance proteins.

In summary, we describe a sensitive mass spectrometric method that allows generating highly accurate and reproducible protein quantification data from minute amounts of highly homogeneous cell subpopulations enriched by FACS. This technology allows dissecting the biochemical processes in play in specific cell subpopulations of interest with unprecedented sensitivity, depth of coverage, and reproducibility. Thereby, it paves the way for global proteomic analyses in clinically highly relevant but numerically scarce cell populations such as (pre)leukemic stem cells in hematopoietic malignancies as well as cancer stem cells from solid tumors.

## DATA AVAILABILITY

The mass spectrometric DDA and DIA raw data files and associated search results of the HEK peptide dilution series have been deposited to the ProteomeXchange Consortium (http://proteomecentral.proteomexchange.org) via the PRIDE partner repository (75, 76) with the dataset identifier PXD009246. The mass spectrometric DIA raw data files and associated search results of the CD34+ FACS-isolated dilution series and FACS-isolated stem and progenitor cells have been deposited with the dataset identifier PXD009255. RNA sequencing data have been deposited in NCBI's Gene Expression Omnibus (GEO) (77) (https://www.ncbi.nlm.nih.gov/geo/) with the accession number GSE113182.

## Supplementary Material

ProteinQuantification_HSPC

Supplemental Data

Supplemental Table S5

Supplemental Table S6

## Footnotes

^1^ The abbreviations used are:

CMP: common myeloid progenitors
CV: coefficient of variation
DDA: data-dependent acquisition
DIA: data-independent acquisition
FDR: false discovery rate
GMP: granulocyte-macrophage progenitors
GO: gene ontology
HSC: hematopoietic stem cells
HSPC: hematopoietic stem and progenitor cells
MEP: megakaryocyte-erythrocyte progenitors
MPP: multipotent progenitor cells.
