# Supplementary material for: Sensitive Quantitative Proteomics of Human Hematopoietic Stem and Progenitor Cells by Data-independent Acquisition Mass Spectrometry
Source: Mol Cell Proteomics. 2019 Apr 11;18(7):1454–67. doi: 10.1074/mcp.TIR119.001431 (PMC6601215; doi:10.1074/mcp.TIR119.001431)
Supplement: Supplemental Data [file 144140_1_supp_312069_ppnkkc.pdf]

Supplemental Data for

Sensitive quantitative proteomics of human hematopoietic stem and progenitor cells by data-independent acquisition mass spectrometry

Sabine Amon*, Fabienne Meier-Abt*, Ludovic C Gillet, Slavica Dimitrieva, Alexandre PA Theocharides, Markus G Manz, and Ruedi Aebersold

*These authors contributed equally to this work.

Ruedi Aebersold
Email: aebersold@imsb.biol.ethz.ch

This PDF file includes:

Supplemental text
Figs. S1 to S19
Tables S1 to S4
References for supplemental data

Other supplemental materials for this manuscript include the following:

Datasets S5 and S6: GO gene sets

Supplemental Text

Optimization of DIA-MS for small sample loads. Two types of instrument platforms have been used to acquire DIA data, QqTOF and Orbitrap instruments. They fundamentally differ in the way they transmit ions and acquire mass spectra. QqTOF instruments are typically operated with fixed tandem mass spectra (MS2) acquisition times and therefore duty cycles. Because ions are transmitted to the detector without any accumulation step, this type of instrument offers relatively few parameters for fine tuning. In contrast, newer generation instruments equipped with Orbitrap analyzers allow the optimization of data acquisition at several stages, e.g. by varying the scan time (resolution is directly proportional to the detection time of the transient), by optimizing the number of accumulated target ions and/or the maximum accumulation (injection) time prior to the detection event, and finally by executing accumulation and detection steps in parallel, as implemented on the Lumos instrument. Depending on the intended application and the available sample amounts, optimization of these parameters may yield substantial gains in performance.

We therefore studied the dependency of fill times to reach a desired ion population on the amount of tryptic peptides from a HEK293 cell lysate loaded on column. We used a 40 DIA isolation window scheme spanning 400-1000 m/z that showed optimal identification results for 500 ng peptide load, in combination with a 2 h long chromatographic gradient (data not shown). The data confirm (supplemental Fig. S4) that, as expected, the time required to reach the target value of accumulated ions varied considerably per scan. For the peptide-rich regions of the retention time (RT) vs. m/z graph, specifically between 400-800 m/z and a RT range of 10-80 min, the target was reached within a few ms on average at the highest sample loads. Thus, the accumulation time was much lower than the actual scan time of 64 ms (to reach 30,000 resolution at m/z 200). To optimize the use of ions within the time constraint of each Orbitrap scan event (ions for the next scan are accumulated in parallel to the fragment ion acquisition of the current scan), we selected a scheme that took advantage of a median injection time of 30-50 ms (supplemental Fig. S4B). At 250 ng sample load this already corresponded to a six- to nine-fold increase of the fill time compared to a 2 µg sample load (supplemental Fig. S4C). To assess the performance limits of the optimized acquisition scheme, we compared the number of identified protein groups of a standard data-dependent acquisition (DDA) method to the optimized DIA protocol with triplicate injections of a dilution series (in steps of ½) of HEK293 peptides ranging from 2 µg to 3.9 ng. The results indicate that the DIA mode systematically identified a higher number of peptide precursors and protein groups than DDA for sample loads above 30 ng (Fig. 1A and supplemental Fig. S5). The average number of identified protein groups decreased by only 12% (from 7,406 to 6,472 applying a precursor Q- value cutoff of 0.01 (see Experimental Procedures)) in DIA when reducing the sample amount from 2 µg to 125 ng of peptide mass on column. For the same concentration range, the fraction of protein groups consistently identified in all three injections remained above 85% in DIA (supplemental Fig. S6A and B, Table S4) and >98% of protein groups

identified at low sample loads were also found with the highest loads (supplemental Fig. S6C).

Next, we assessed the quantitative reproducibility and accuracy of the dilution series data generated in DIA mode. An average peptide quantification coefficient of variation (CV) of less than 10% was obtained for triplicate injections across the whole dilution series, even at a level of 3.9 ng (Fig. 1B). The run-to-run correlation coefficient was above 0.96 throughout the dilution series (supplemental Fig. S7). The peptide quantification values obtained for the consecutive dilution steps retained linearity throughout the entire dilution range (Fig. 1C), and deviated by less than 20% in accuracy compared to the values at the highest sample load. It is noteworthy that for lower sample amounts (<125 ng) we observed a reduction in signal intensity for late eluting hydrophobic peptides that was over proportional to the expected dilution effect (supplemental Fig. S8). We attribute this to adsorption effects (e.g. to the walls of the sample tubes). To maintain sufficient robustness for the measurement of all peptides, irrespective of their hydrophobicity, we therefore decided to set 100-200 ng of peptides as the practical lower limit for the subsequent measurements.

To adapt the sample workup procedure for the low number of sorted cells required to yield the 100-200 ng of peptide mass per DIA measurement we devised a single tube procedure that minimized sample losses (supplemental Fig. S3A). We identified several steps that were critical for the generation of high quality peptide samples, including: (i) FACS in the absence of fetal bovine serum (FBS), (ii) the use of low protein-binding plastic-ware to minimize the loss of hydrophobic peptides, (iii) careful pelleting of the non-adherent sorted cells, (iv) freeze-drying of the pellets and subsequent lysis and digestion in the smallest volume that can be handled practically (supplemental Fig. S3B-C and expanded details in Experimental Procedures).

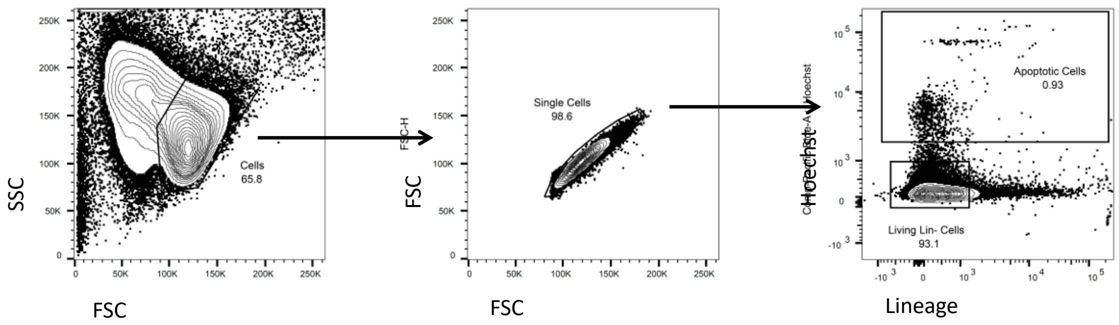

Fig. S1. The proportion of apoptotic cells as determined by the proportion of Hoechst positive cells lay below 1%.

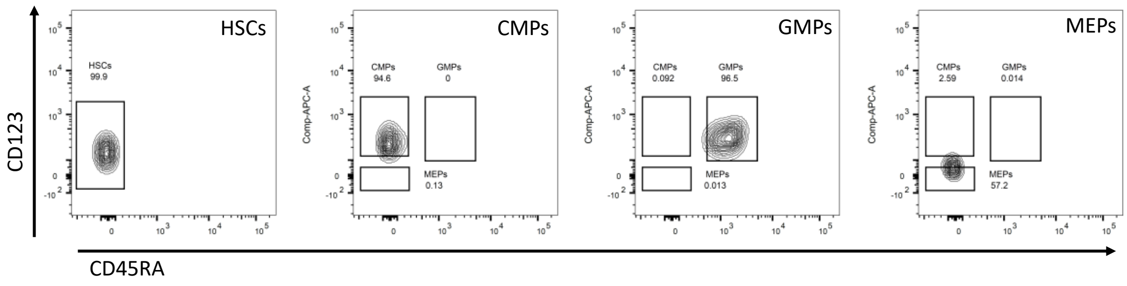

Fig. S2. Homogeneity of cell subpopulations obtained by FACS. Reanalysis of sorted populations showed spill-over into other gates of less than 3% for highly enriched HSCs/MPPs (referred to as HSCs), CMPs, GMPs and MEPs.

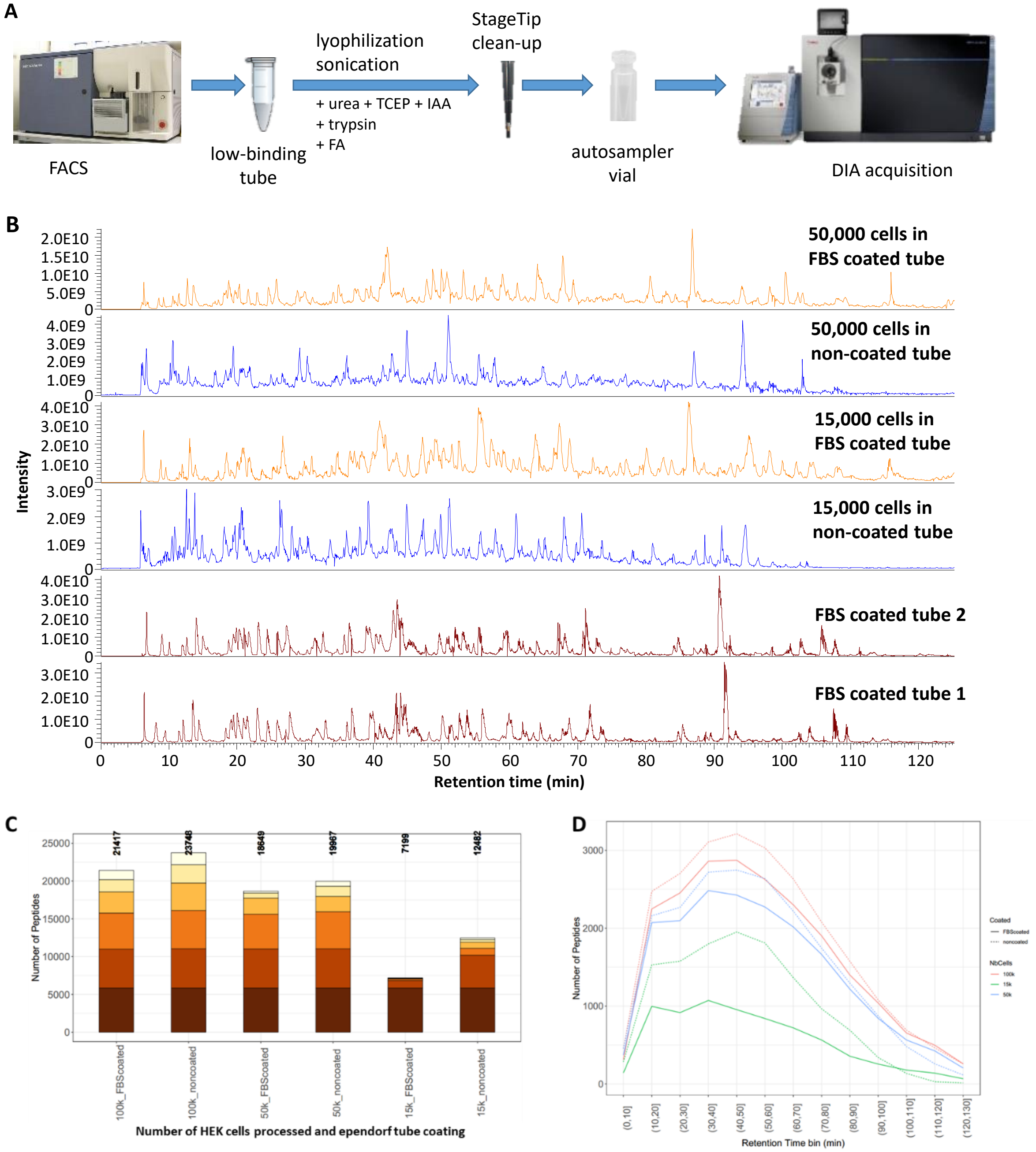

continued on next page

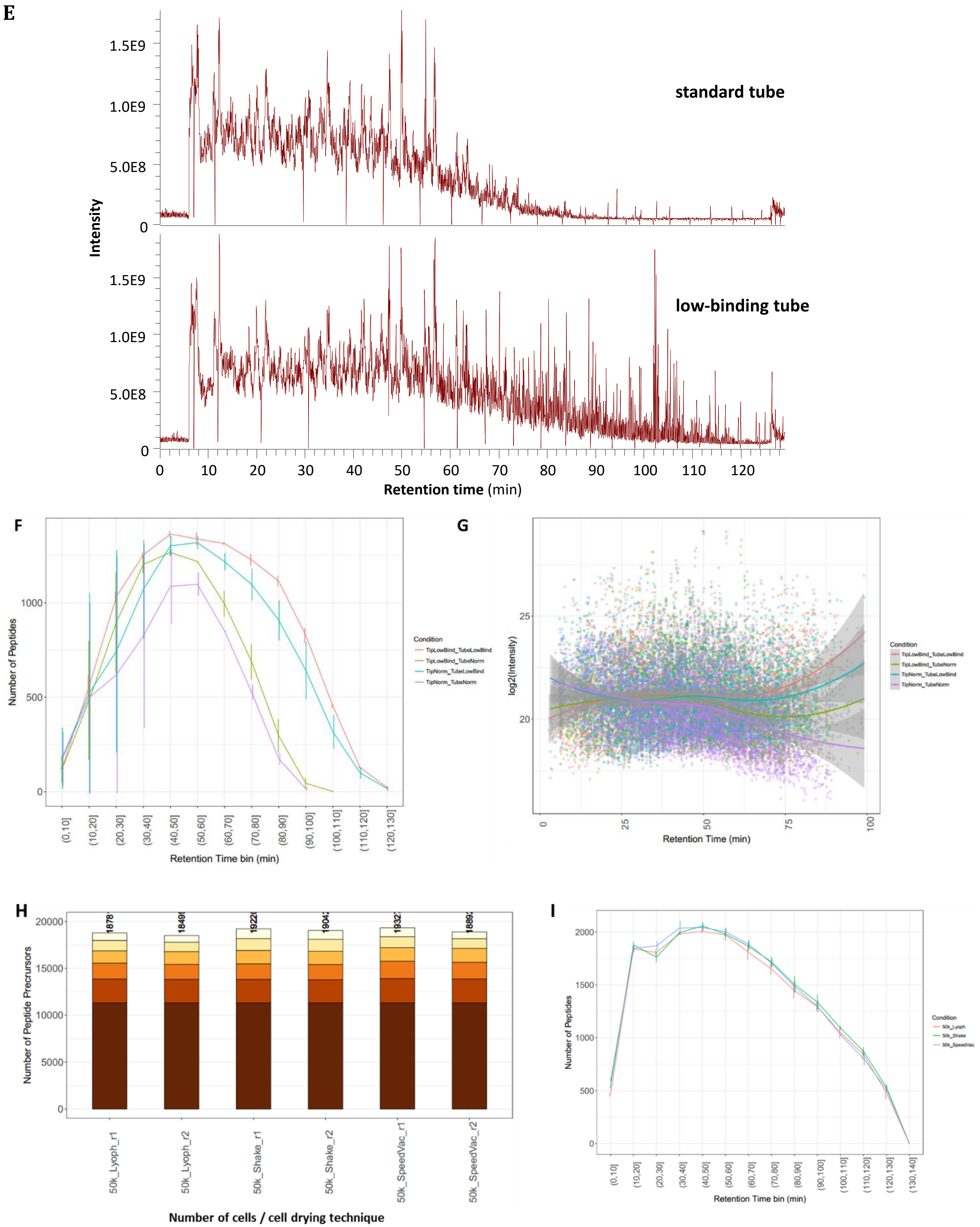

continued on next page

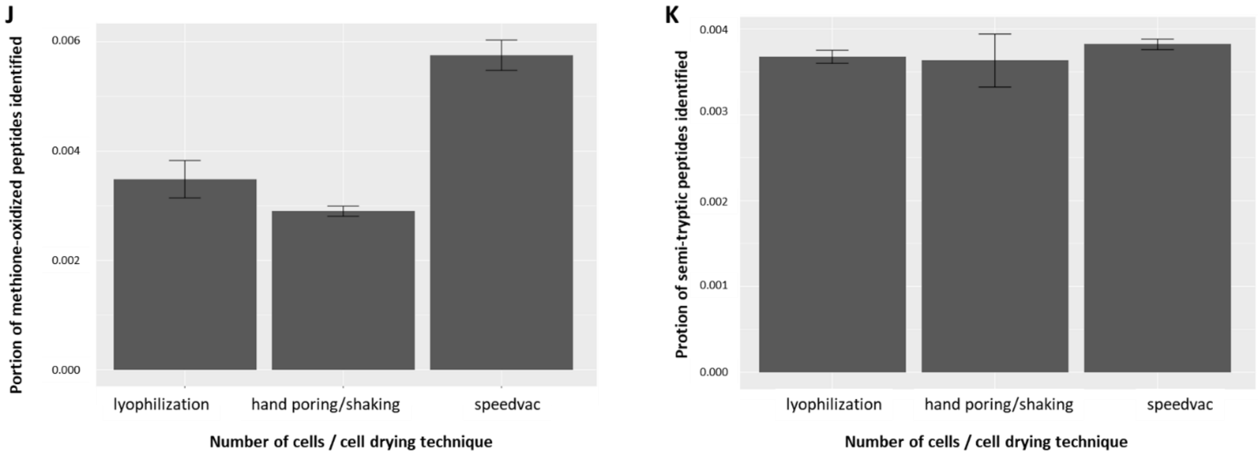

Fig. S3. Critical steps for miniaturized sample handling. (A) Experimental workflow optimized for collecting and processing the low cell numbers analyzed in the study: Cells were sorted into a low-protein-binding micro-centrifuge tube and pelleted. The supernatant was removed very carefully, leaving approx. 50 µl of buffer to not disturb the cell pellet. The sample was then lyophilized and the pellet was re-suspended in 10 µl of lysis buffer. Following reduction, alkylation, and trypsin digestion, the sample was transferred to a StageTip for desalting. The eluted peptides were vacuum dried, re-suspended in 10 µl of LC solvent A with the addition of iRT peptides and transferred into an autosampler vial for LC-MS/MS analysis. (B-D) FBS tube coating strongly increases background. FACS-isolated CD34+ hematopoietic stem/progenitor cells were collected either in FBS pre-coated or non-coated low-binding micro-centrifuge tubes. Additionally, FBS pre-coated low-binding micro-centrifuge tubes were processed without added sample cells. The respective total ion chromatograms are shown (B) as well as the corresponding number of peptide identifications overall (C) or in 10 min bins (D). In our hands, the FBS coating systematically decreased the number of DIA peptide identifications overall, without any obvious bias towards peptide hydrophobicity. These results somewhat contradict the BSA-coating recommendation of *Kasuga, K., et al. (2017) Proteomics 17*. This could be explained by the limitations of DIA in analyzing samples with very large dynamic range: because of the larger precursor isolation windows used in DIA vs DDA, the most intense peptides (typically originating from the coating for those low sample loads) would participate in the ion trap filling to a much greater extent than that of the (very) low endogenous peptide sample signals, causing a challenging intra-scan dynamic range for the analyzer. (E-G) Attrition of hydrophobic peptides in standard compared to low-binding micro-centrifuge tubes. A HEK293 peptide stock solution of 1 µg/µl was diluted 1/40 either in standard or in low-binding micro-centrifuge tubes and transferred to autosampler vials after 30 min. 4 µl (corresponding to 100 ng of peptides) were analyzed in DDA mode. The respective total ion chromatograms are shown (E). The number of identified protein groups and peptides decreased by 25% and 43%, respectively, for the peptide solutions being diluted in standard micro-centrifuge tubes in comparison to low-binding tubes. The data shows a specific attrition of identification for the late eluting, more hydrophobic, peptides (F). The effect is more obvious when plotting the MS1 intensity of the shared peptides (identified throughout all runs). (G) Low binding tubes systematically yield higher intensities for the late eluting peptides; the effect of the low binding tips is less clear: though they seem to improve the number of identifications (F) and the intensity (G) for the late eluting peptides, it seems to come at the cost of the early eluting peptides, whose intensities seem to be lower in the low-binding tips vs normal tips. (H-K) Effects of the lyophilization vs. handporing/shaking vs speedvac sample drying protocols. 50k HEK293 cells were collected in low

binding Eppendorf tubes and the supernatant was then removed either by lyophilization, hand poring/shaking, or speedvac, prior to adding the urea lysis buffer and proceeding to the peptide digestion. The protocols do not show any significant difference in overall number of identification (H), nor any bias in the hydrophobicity of the peptide recovered (I). The hand poring/shaking yields slightly less oxidation on methionine residues and semi-tryptic/hydrolized peptides, but is not amenable to handling low amount of non-adherent cells such as the HSCP cells. The lyophilization constitutes a reasonable compromise.

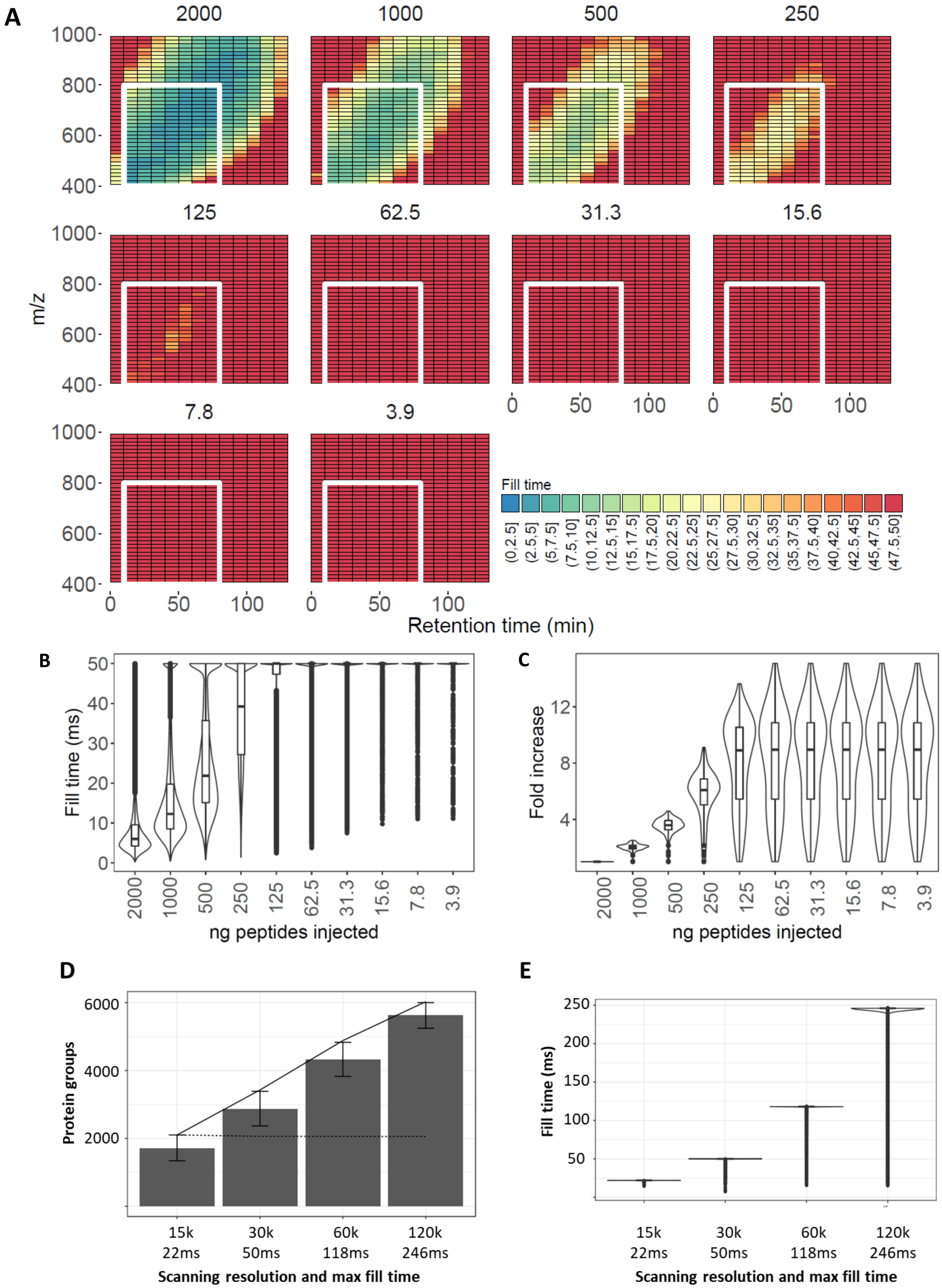

continued on next page

F

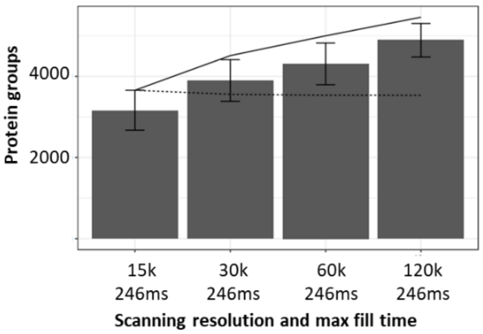

Fig. S4. Fill times for various HEK293 peptide loads. (A) Averaged fill time required to reach the automated gain control (AGC) target. Fill times were averaged in 10 minute retention time (RT) bins for each of the 40 DIA isolation windows and are colored using a color scale from red for the bins with the longest fill times (i.e., 47.5-50 ms) to blue for the bins with the shortest fill times (i.e., 0-2.5 ms). The white rectangle indicates the peptide-rich region between 400-800 m/z and RT 10-80 min that was used for the violin plots in B and C. (B) Distribution of the fill times for the region highlighted in A. (C) Violin plot showing the increase in fill time upon serial dilution compared to that observed at a sample load of 2000 ng which is typical for DIA analyses. (D-E) Number of protein groups identified (D) and corresponding experimental fill time (E) at 31.3 ng load of HEK293 tryptic peptides upon increasing Orbitrap scanning resolution and fill time setups. To maintain a comparable cycle time between those datasets, we only covered the mass space from 535 to 685 m/z only using 11 precursor isolation windows of 15 Da, with the rest of the cycle time being spent acquiring a set of 1-Da windows at mass 1500m/z (70, 30, 10 or 0 times for the 15k, 30k, 60k and 120k resolution methods respectively), not used in that analysis. (F) Number of protein groups identified at 31.3 ng load of HEK293 tryptic peptides upon increasing Orbitrap scanning resolution and a fixed fill time set to 246 ms. To be comparable with (D), we again only covered the mass space from 535 to 685 m/z using 11 precursor isolation windows of 15 Da for those experiments. This data shows that, at 30 ng sample loads, longer fill times increase the number of identifications to a greater extent than higher scanning resolution alone. This illustrates the optimal use of DIA performance at very low sample loads when leveraging the fill time capabilities of ion-trap instruments. For figures D&F: The error bars represent the number of protein groups identified in common (intersection) or in total (union) for the technical triplicate injections at the corresponding peptide load. The solid line shows the cumulative protein group identifications across all peptide loads, the dotted line the shared protein group identifications across all peptide loads.

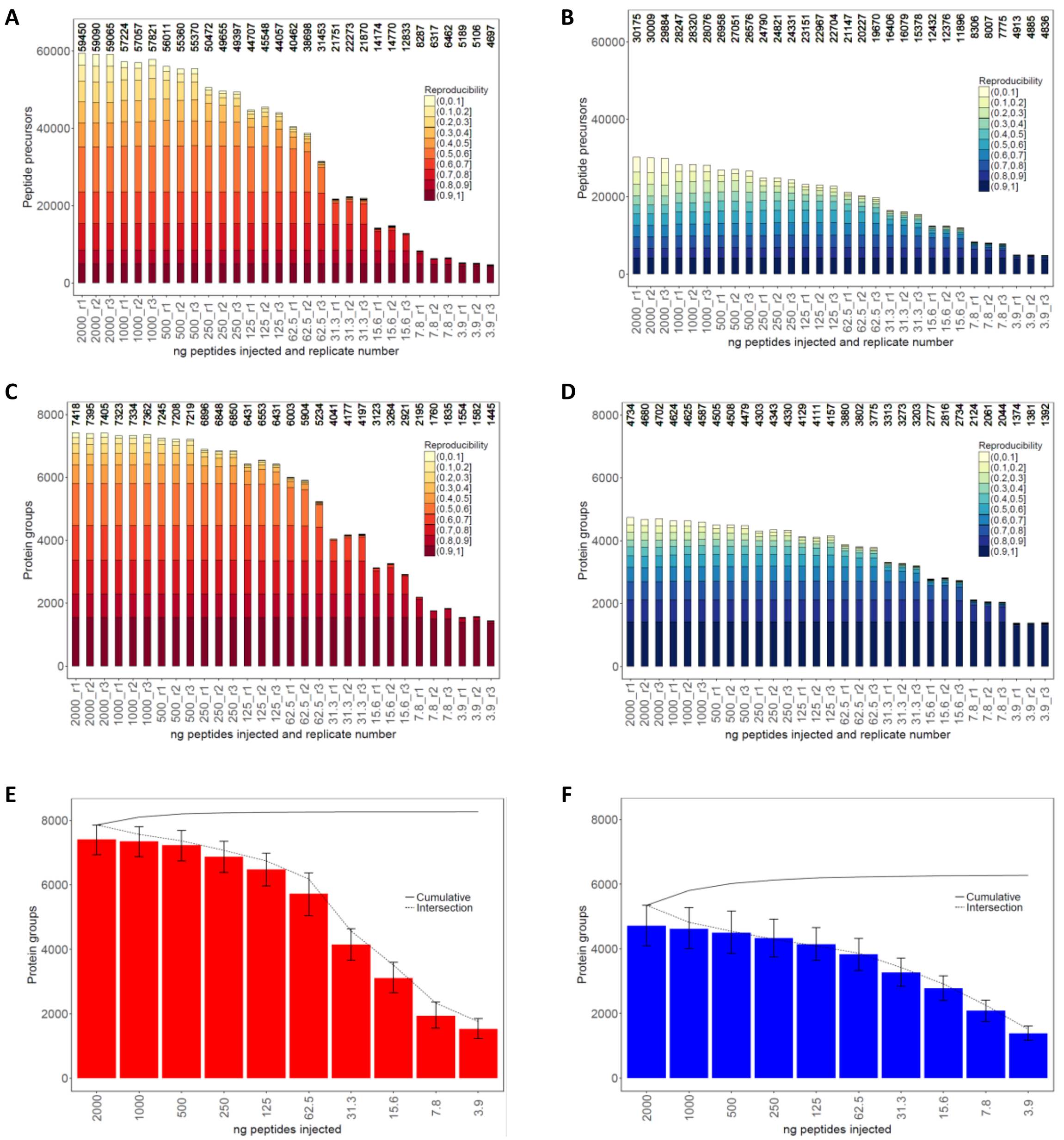

Fig. S5. Number of peptide precursors (A, B) and protein groups (C, D), respectively, identified at 1% peptide FDR in DIA (A, C) and DDA (B, D) mode for decreasing loads of HEK293 tryptic peptides. The reproducibility refers to the fraction of runs in which a particular peptide precursor or protein group was identified. All numbers referred in this figure and in all other figures hereafter are specified at 1% peptide FDR except when otherwise explicitly mentioned. Number of protein groups identified in DIA (E) and DDA (F) mode for decreasing loads of HEK293 tryptic peptides. The error bars represent the number of protein groups identified in common (intersection) or in total (union) for the technical triplicate injections at the corresponding peptide load. The solid line shows the cumulative protein group identifications across all peptide loads, the dotted line the shared protein group identifications across all peptide loads.

A

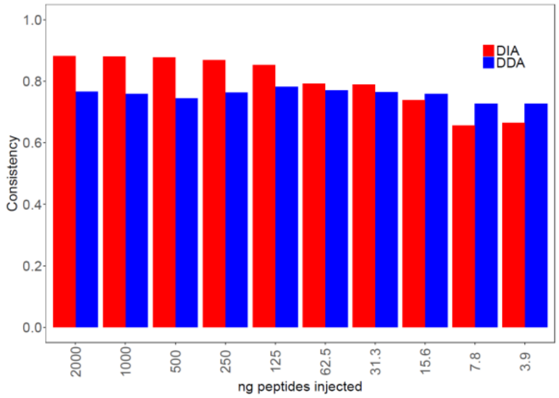

B

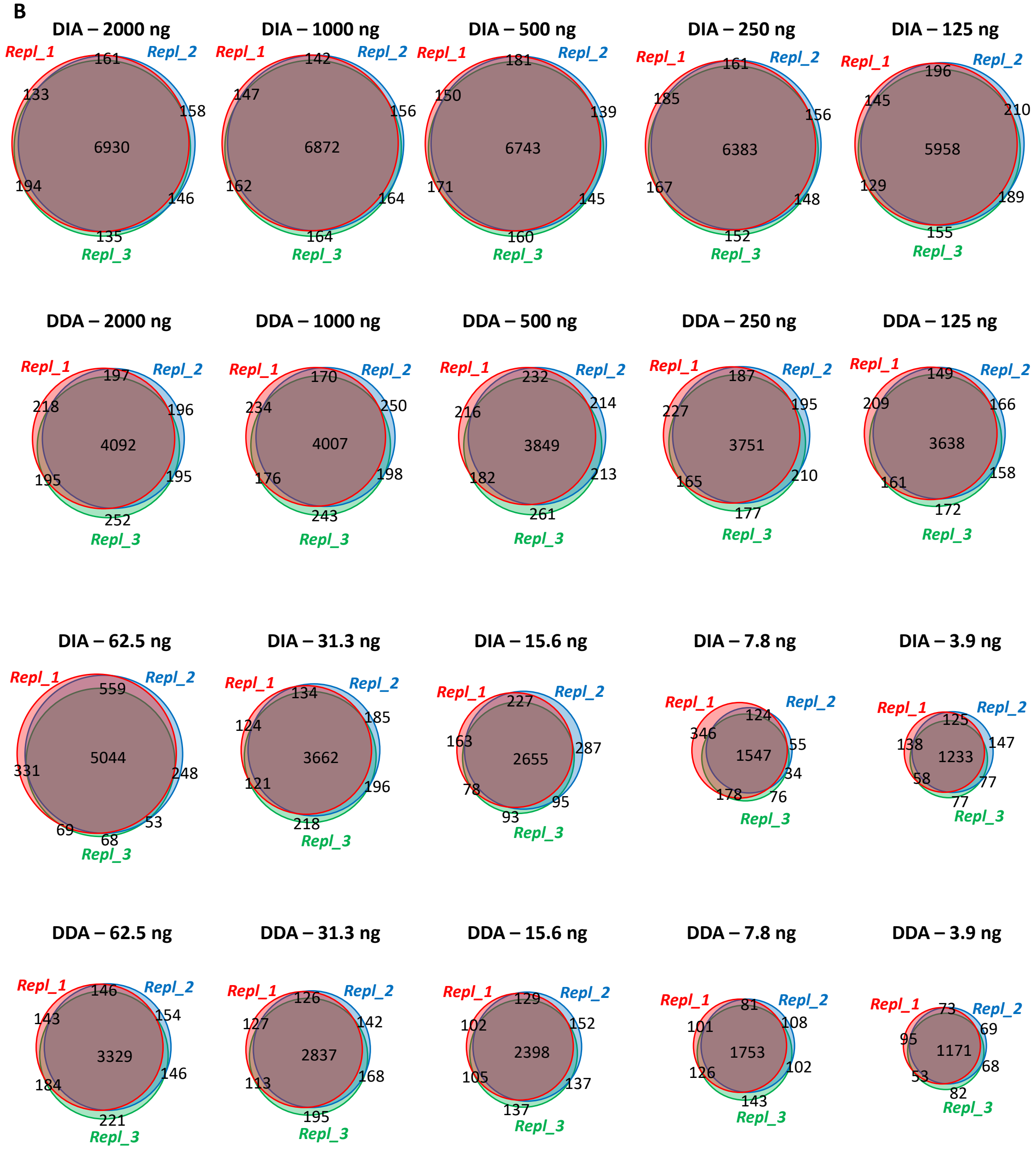

continued on next page

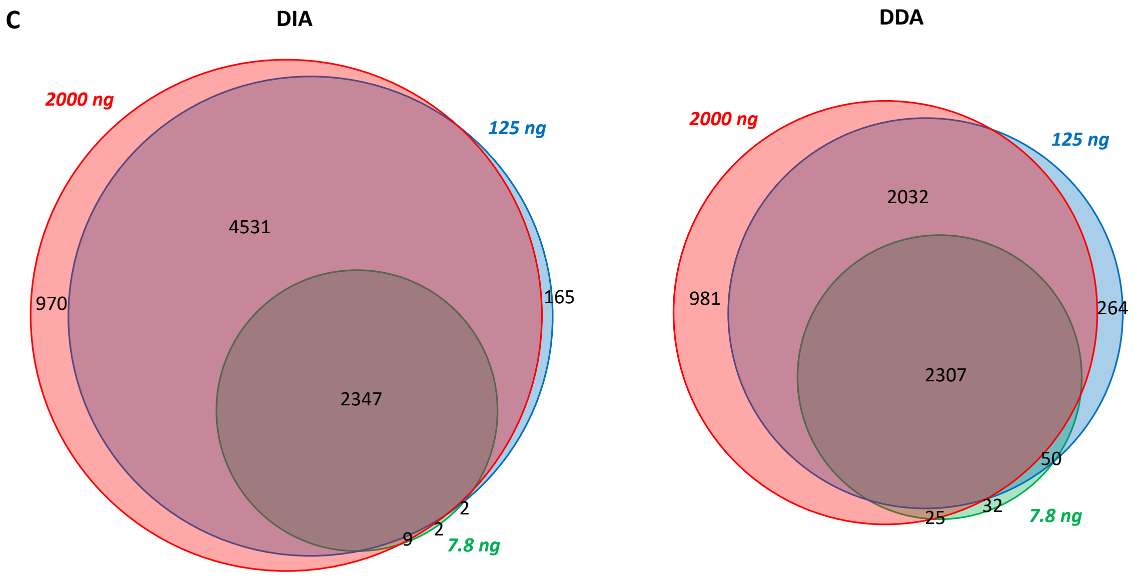

Fig. S6. Consistency of identified protein groups. (A) Consistency of protein group identifications in DIA (red) and DDA (blue) mode, respectively, for the technical replicate injections at various HEK293 tryptic peptide loads. The consistency is calculated as the number of protein groups commonly identified for the triplicate injections divided by the number of protein groups cumulatively identified for the triplicates. (B) Consistency of protein group identifications in DIA and DDA mode (top and bottom panel, respectively) for the technical replicate injections at decreasing HEK293 tryptic peptide loads (2000 ng – 3.9 ng). (C) Consistency of protein group identifications in DIA and DDA mode (left and right panel, respectively) for technical replicate injections at decreasing HEK293 tryptic peptide loads (2000 ng vs. 125 ng vs. 7.8 ng).

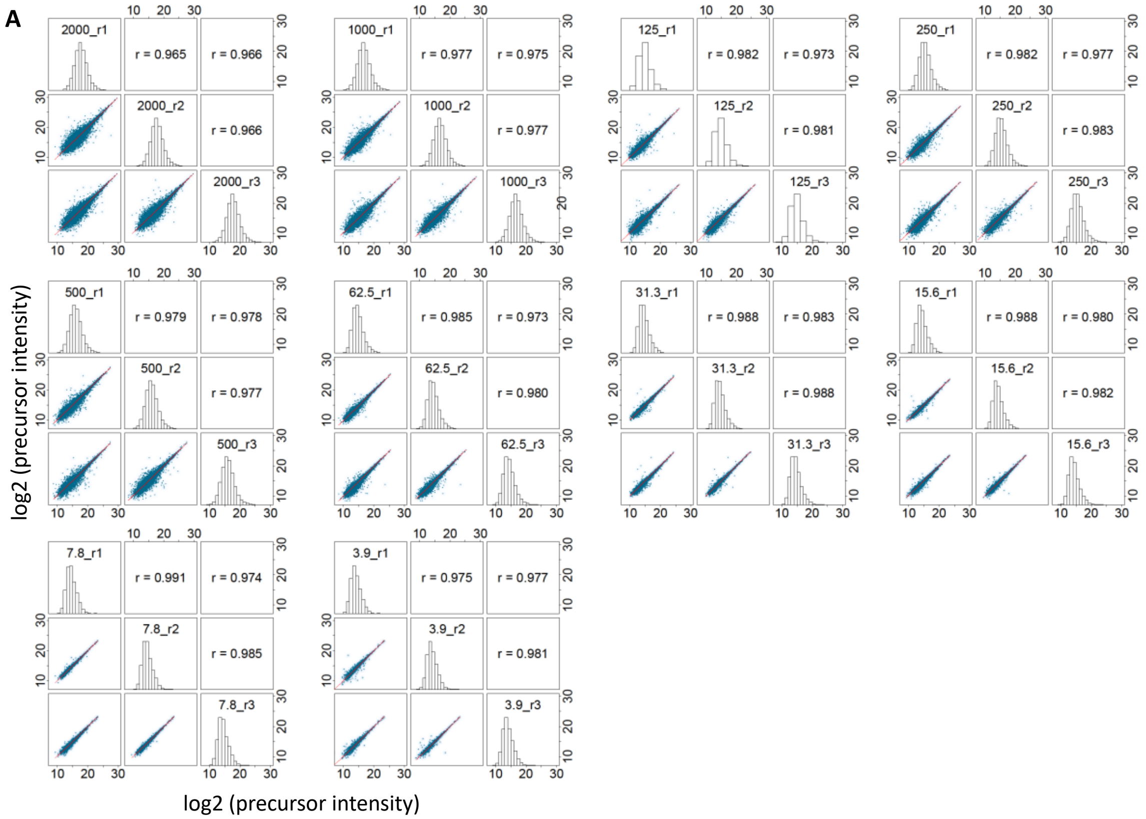

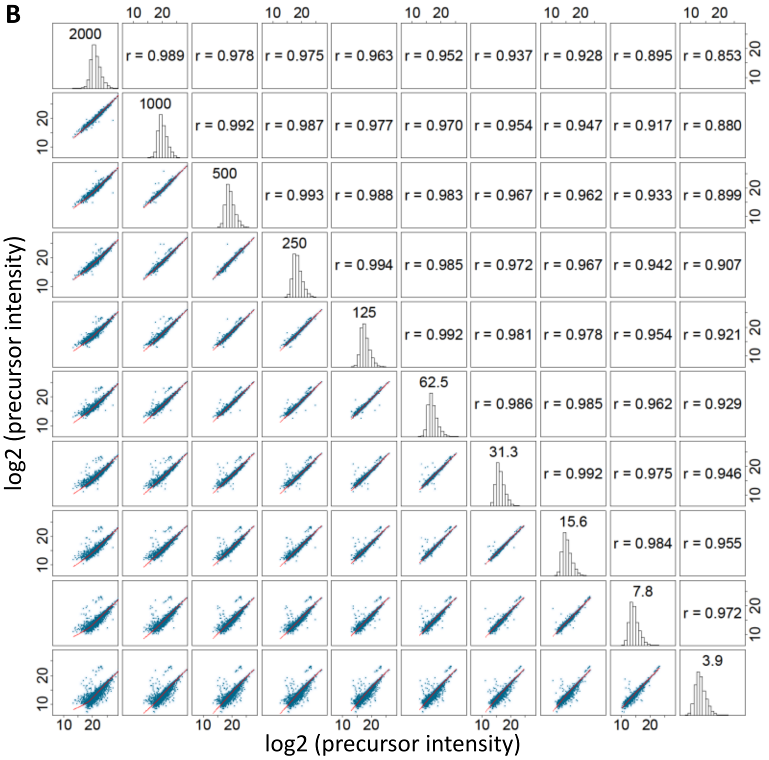

continued on next page

Fig. S7. Intensity correlation across the HEK293 peptide dilution series. (A) Consistency of peptide quantification in DIA mode for technical replicate injections. The x- and y-axes represent the log2 intensity of the peptide precursors for replicates; the Pearson correlation for the scatterplot is shown in the corresponding opposite cell of the matrix. The respective peptide amounts are indicated on top of the bar charts. (B) Consistency of peptide quantification (averaged across the technical triplicate injections of each dilution) for the range of HEK293 tryptic peptide loads (2000 ng – 3.9 ng, as indicated on top of the bar charts); the x- and y-axes represent the log2 intensity of the peptide precursors between the compared dilutions; the Pearson correlation for the scatterplot is shown in the corresponding opposite cell of the matrix.

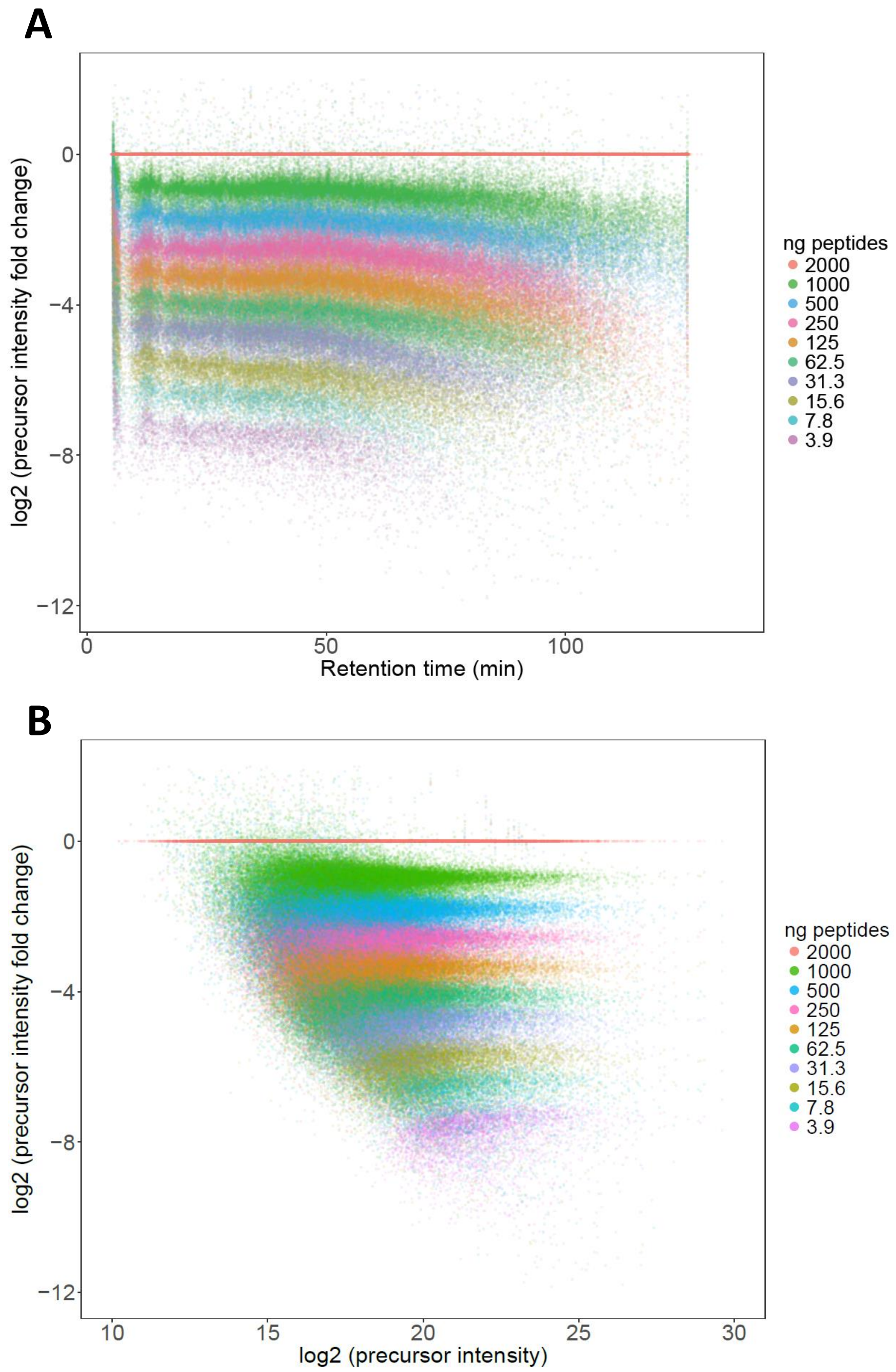

Fig. S8. Spread of the fold changes (in log2 scale) of average peptide precursor intensities reported for a given sample load compared to the 2000 ng sample load. The changes are plotted either (A) against the averaged retention time of the peptide precursors detected at 2000 ng sample load, or (B) against the average intensity (in log2 scale) of the peptide precursor detected at 2000 ng sample load.

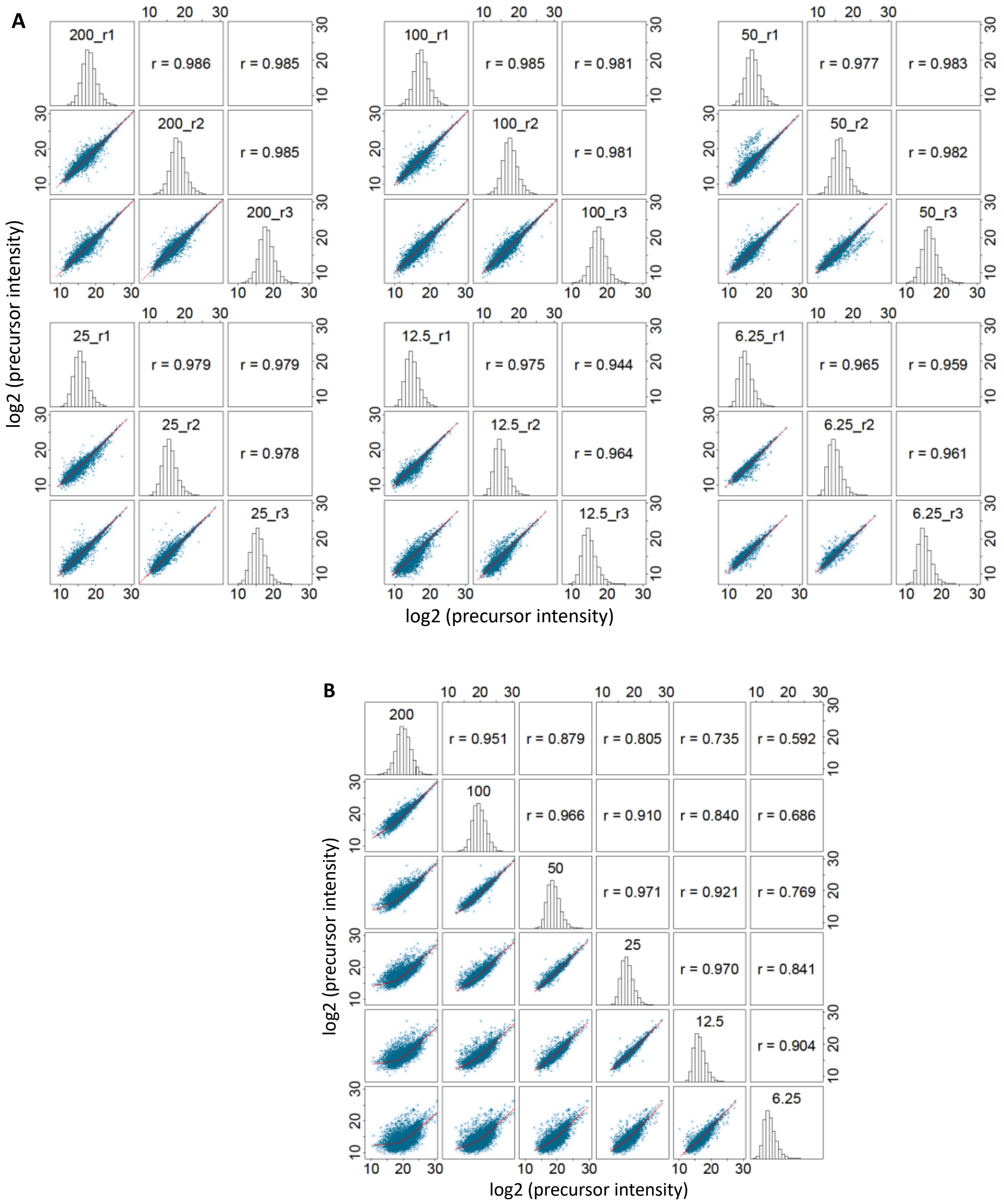

Fig. S9. (A) Consistency of peptide quantification in DIA mode for technical replicate injections of decreasing numbers of FACS-isolated CD34+ hematopoietic stem/progenitor cells (200,000 – 6,250 cells, as indicated on top of the bar charts); the x- and y-axes represent the log2 intensity of the peptide precursors for replicates; the Pearson correlation for the scatterplot is shown in the corresponding opposite cell of the matrix. (B) Consistency of peptide quantification (averaged across the technical triplicate injections of each cell amount) across the various numbers of FACS-isolated CD34+ hematopoietic stem/progenitor cells (200,000 – 6,250 cells, as indicated on top of the bar charts); the x- and y-axes represent the log2 intensity of the peptide precursors between the compared dilutions; the Pearson correlation for the scatterplot is shown in the corresponding opposite cell of the matrix.

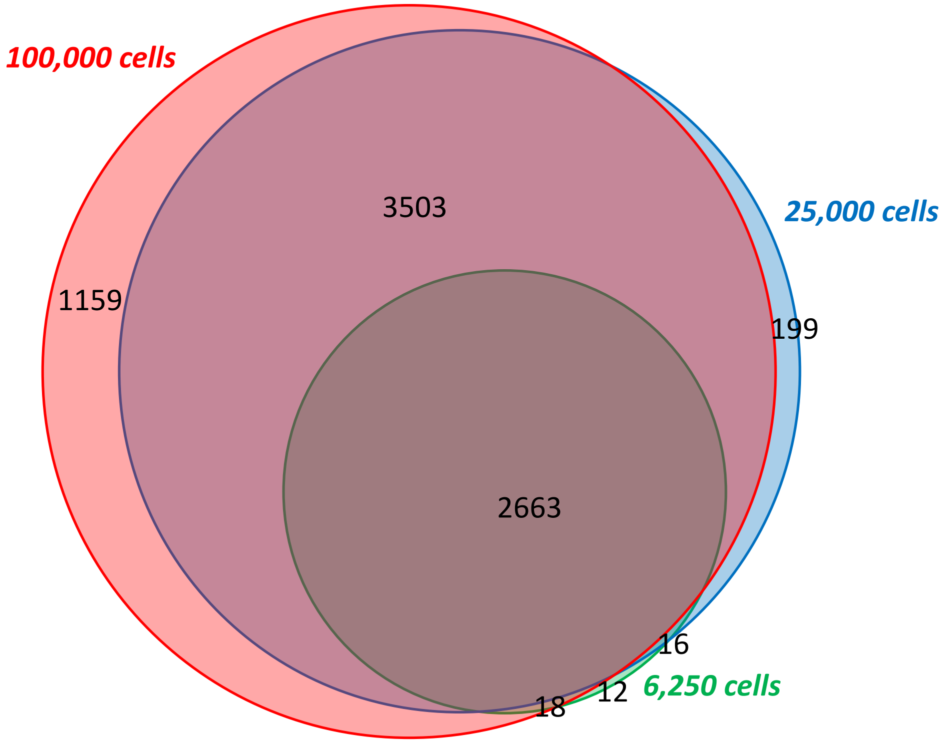

Fig. S10. Consistency of combined protein group identifications in DIA mode for the technical replicate injections between the various CD34+ FACS-isolated cell numbers (100,000 vs 25,000 vs. 6,250 sorted cells).

A

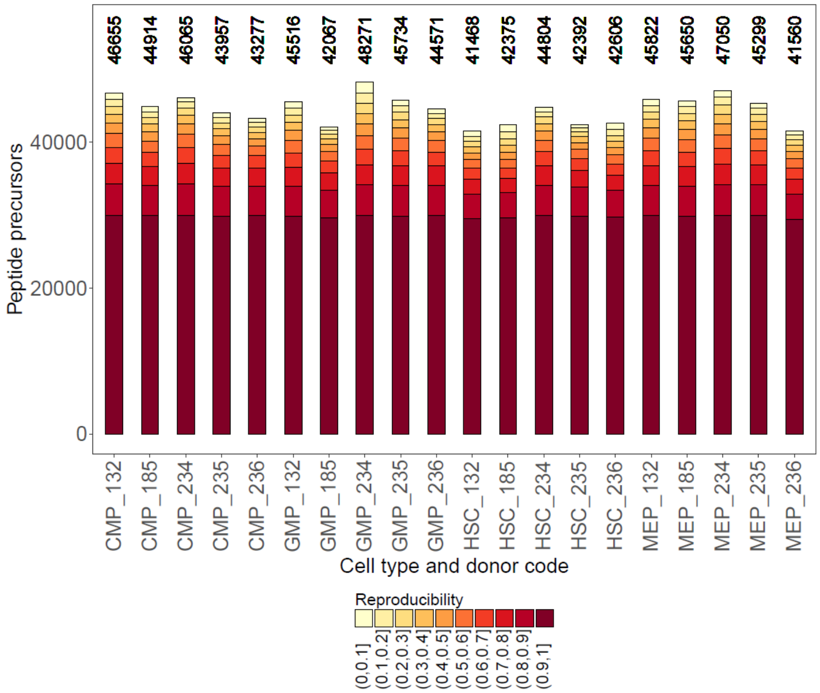

B

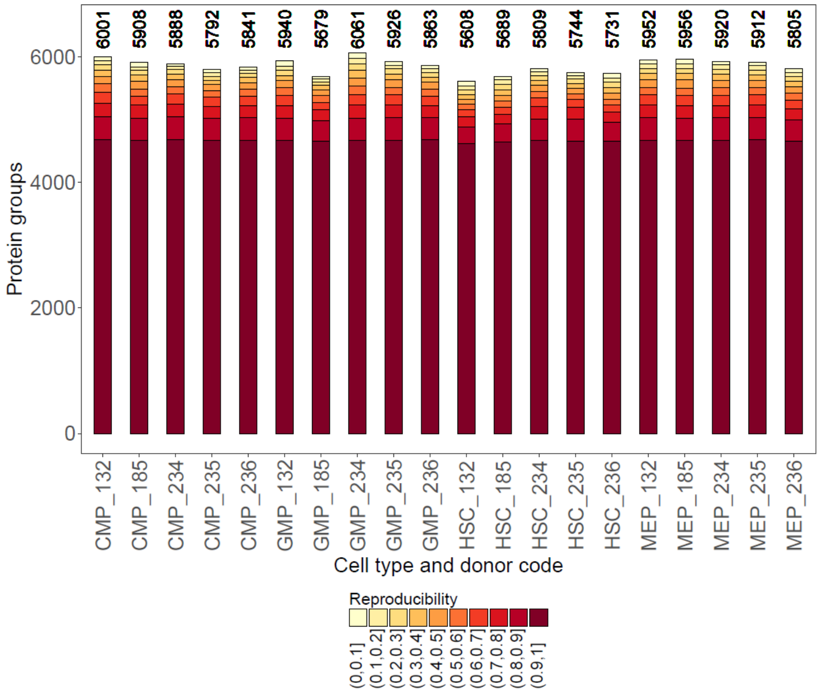

Fig. S11. Number of peptide precursors (A) or protein groups (B) identified in DIA mode for the various series of FACS-isolated hematopoietic stem and progenitor cells. The reproducibility refers to the fraction of samples in which a particular peptide precursor or protein group was identified.

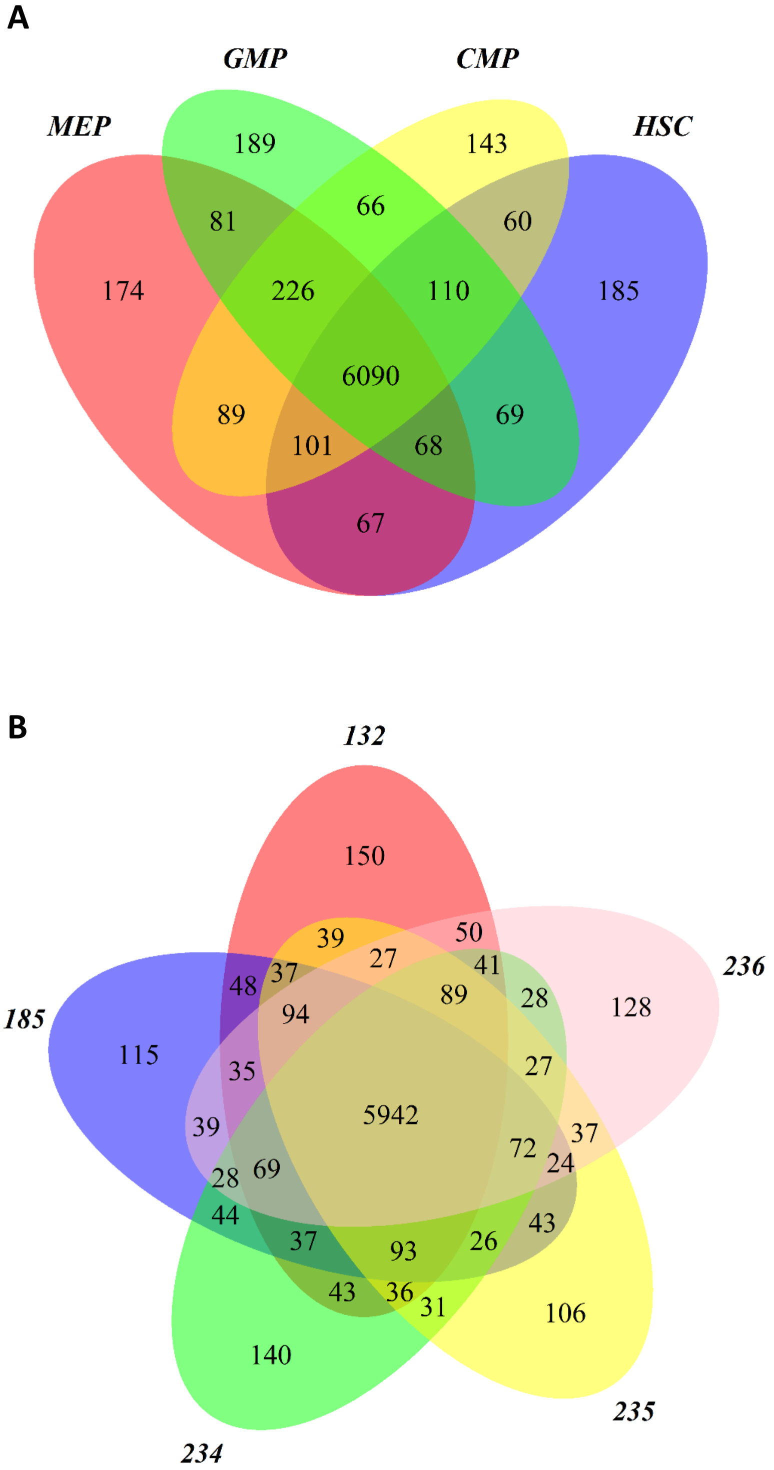

Fig. S12. Consistency of the protein group identification in DIA mode for four FACS-isolated stem and progenitor cell types (MEP, GMP, CMP and HSC) sorted from five donors (donor code 132, 185, 234, 235, 236) plotted (A) according to cell type and (B) according to donor. HSC/MPP is referred to as HSC.

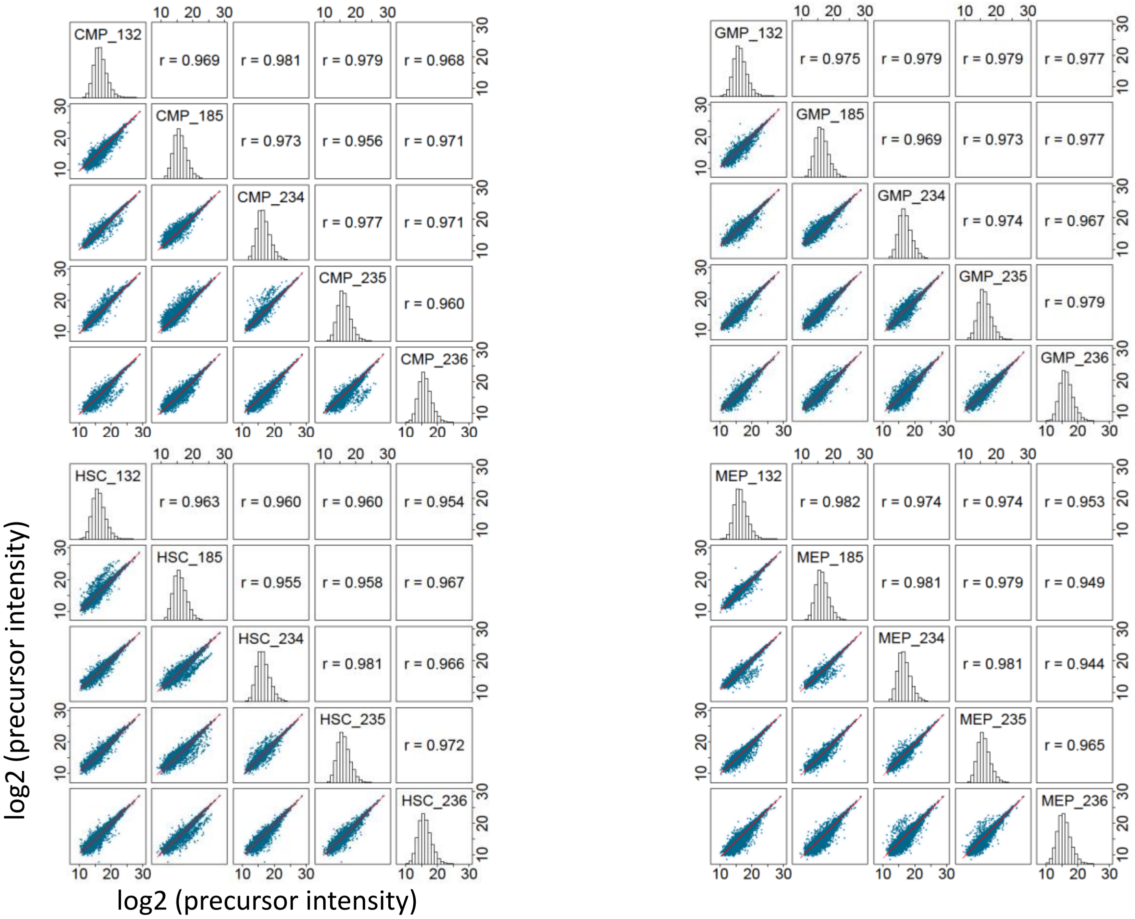

Fig. S13. Consistency of peptide quantification in DIA mode for FACS-isolated stem and progenitor cells (MEP, GMP, CMP and HSC) from five donors (132, 185, 234, 235, 236); the cell type and donor code are indicated on top of the bar charts. The x- and y-axes represent the log2 intensity of the peptide precursors between different donors for each cell type; the Pearson correlation for the scatterplot is shown in the corresponding opposite cell of the matrix. HSC/MPP is referred to as HSC.

A

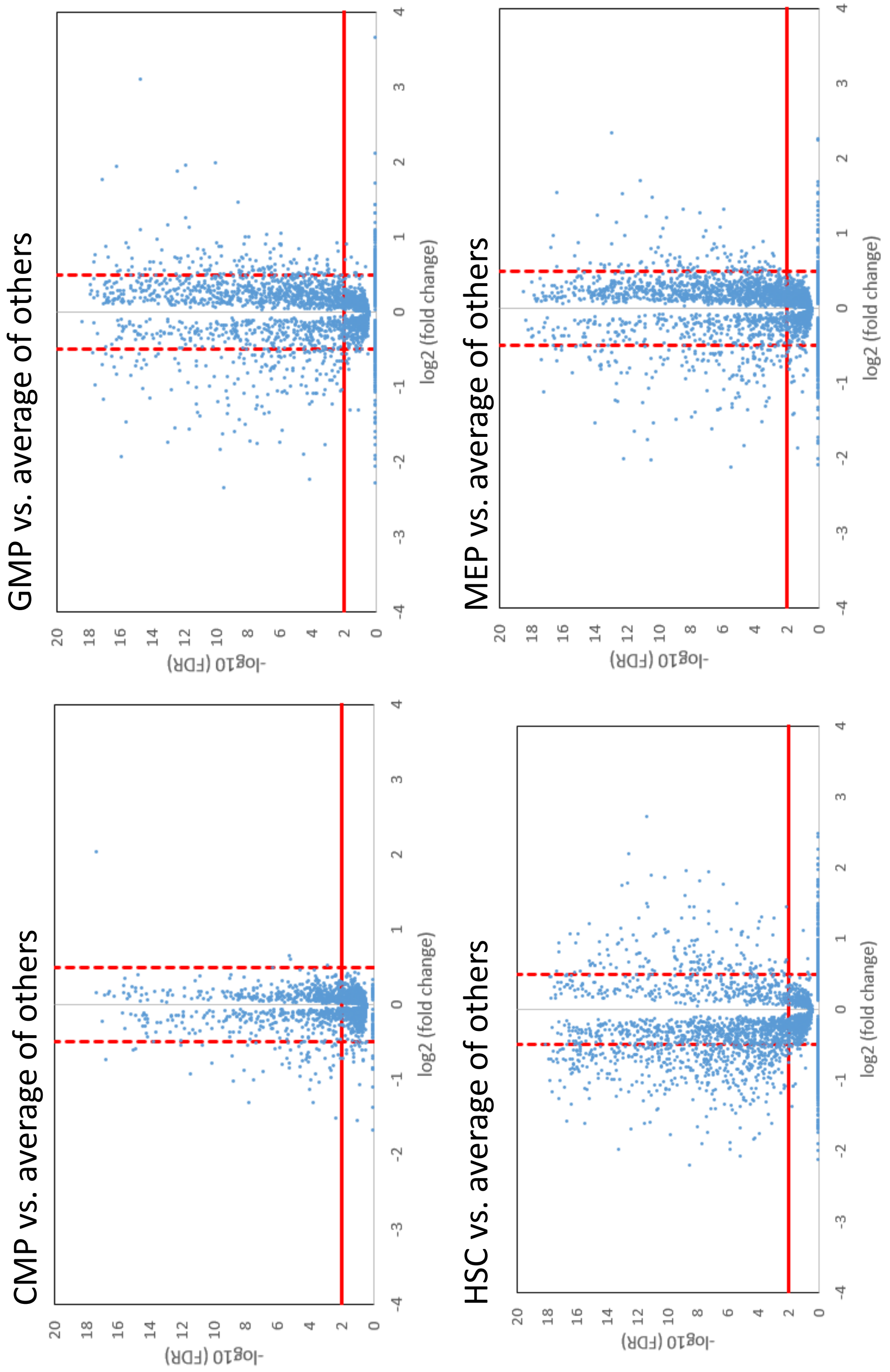
GMP vs. average of others
MEP vs. average of others
CMP vs. average of others
HSC vs. average of others
log2 (fold change)
-log10 (FDR)

B

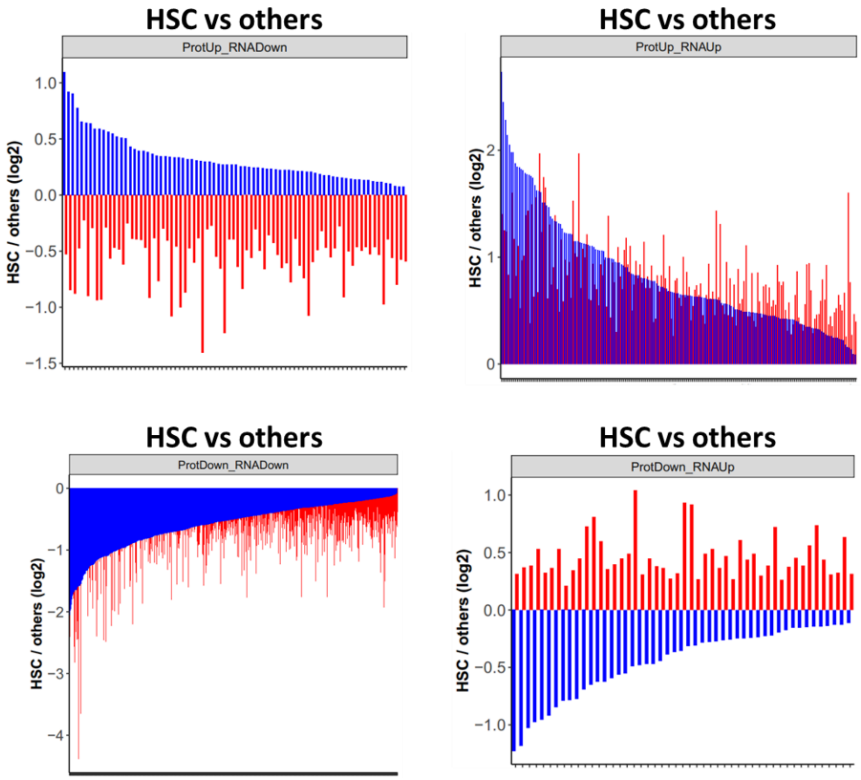
HSC vs others
ProtUp_RNADown
HSC / others (log2)
HSC vs others
ProtUp_RNAUp
HSC / others (log2)
HSC vs others
ProtDown_RNADown
HSC / others (log2)
HSC vs others
ProtDown_RNAUp
HSC / others (log2)

C

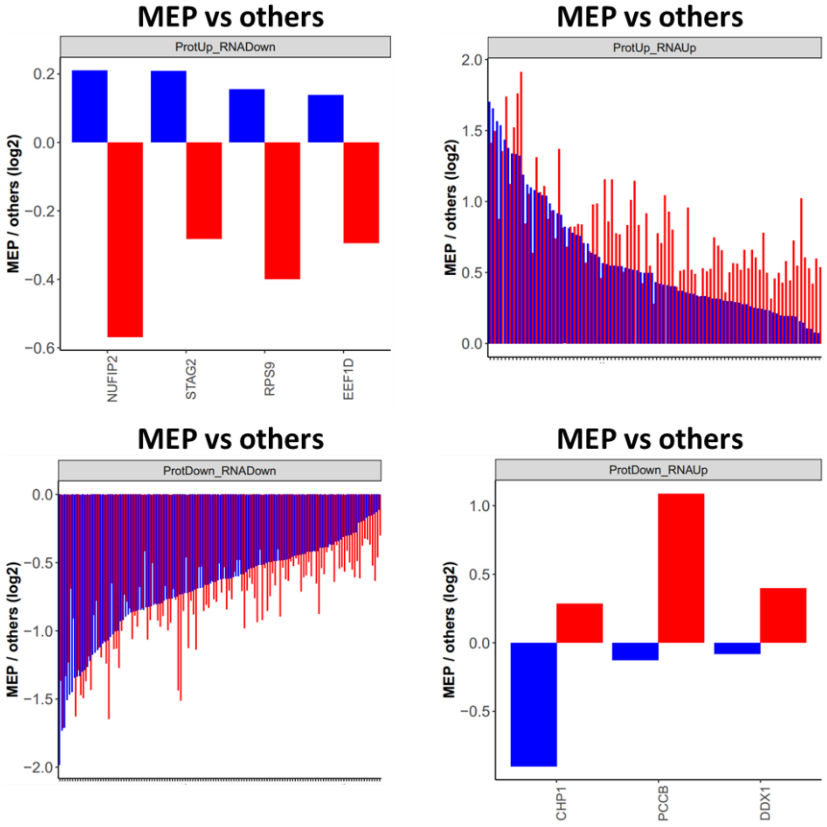
MEP vs others
ProtUp_RNADown
MEP / others (log2)
NUFIP2
STAG2
RPS9
EEF1D
MEP vs others
ProtUp_RNAUp
MEP / others (log2)
MEP vs others
ProtDown_RNADown
MEP / others (log2)
MEP vs others
ProtDown_RNAUp
MEP / others (log2)
CHP1
PCCB
DDX1

D

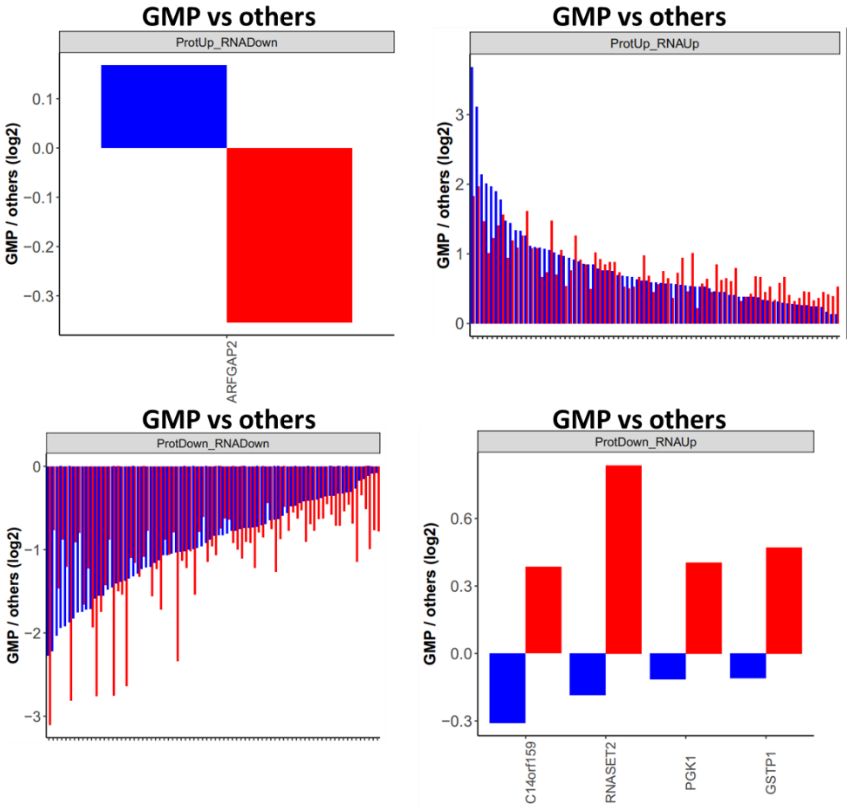

E

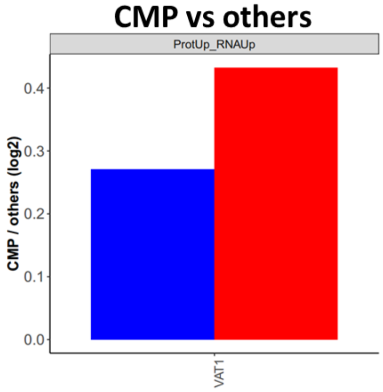

Fig. S14. A. Volcano plots showing differentially abundant proteins when HSCs/MPPs (referred to as HSCs), CMPs, GMPs and MEPs were compared to the average of the other cell subpopulations. Cutoffs of log2(fold change) = 0.5 and of FDR = 0.01 are marked with dashed red and red lines, respectively. **B-E** Bar plots presenting side by side the relative protein (blue bars) and transcript (red bars) fold changes for the protein and transcript presenting significant changes in the cell types HSC, MEP, GMP and CMP respectively.

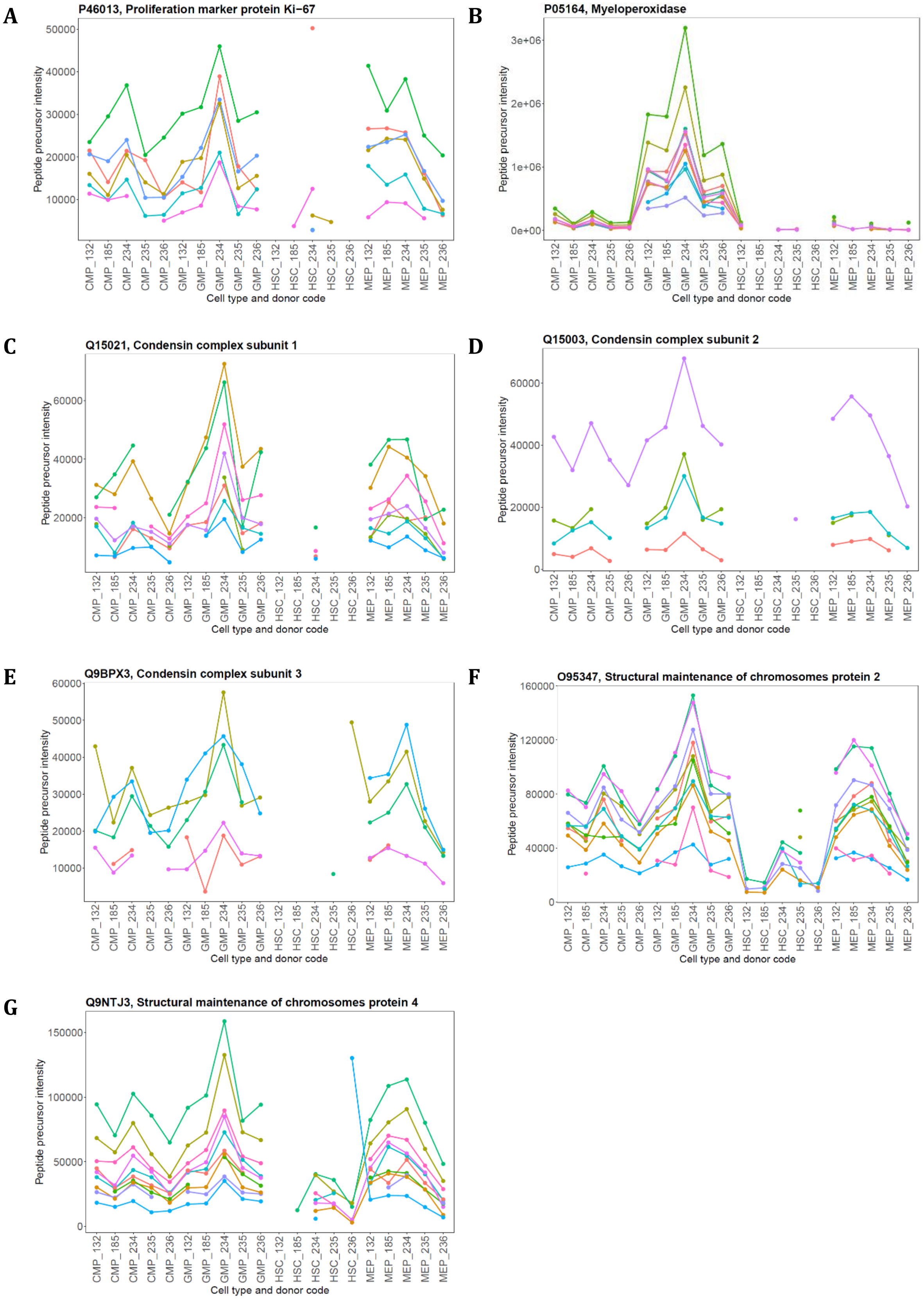

continued on next page

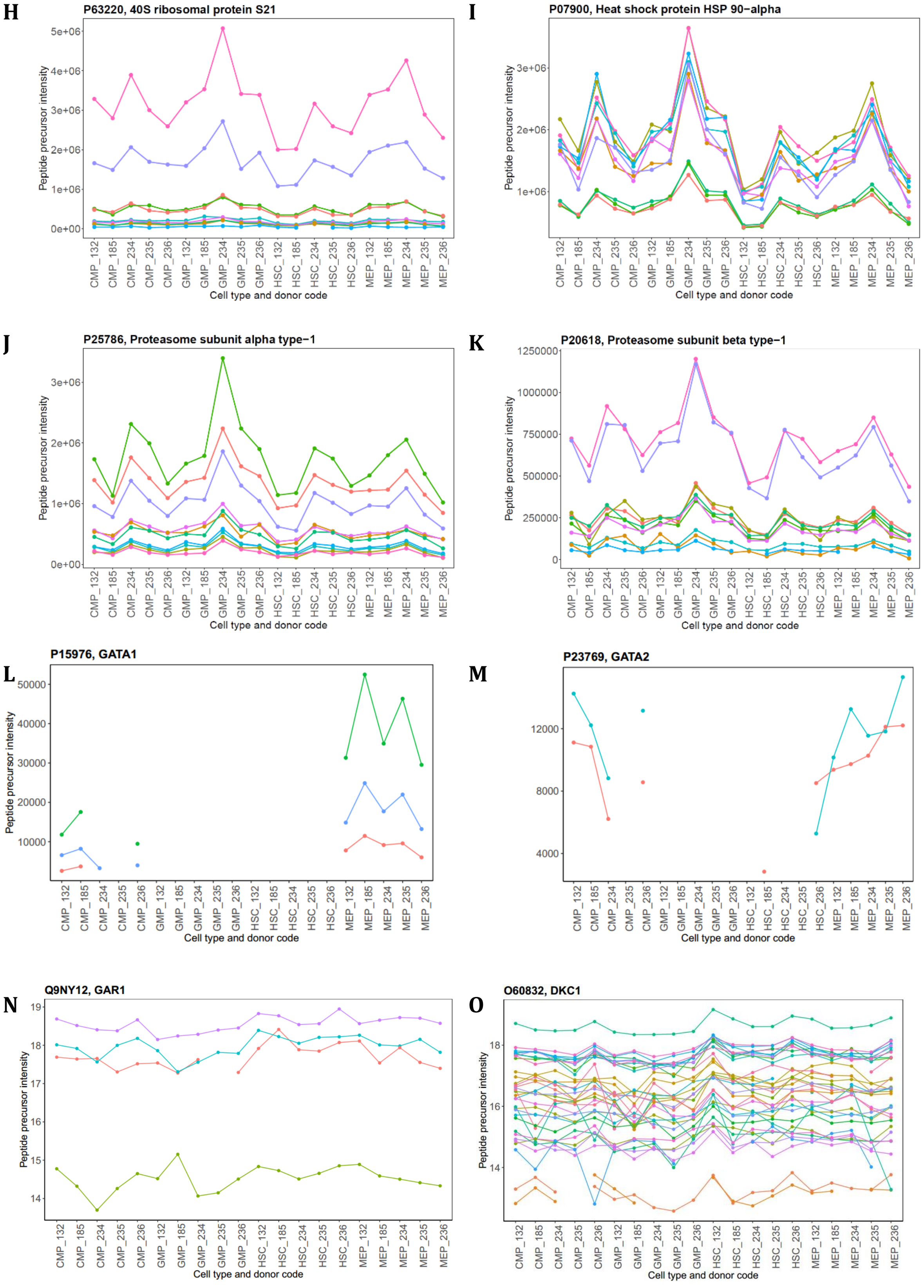
H
P63220, 40S ribosomal protein S21
I
P07900, Heat shock protein HSP 90−alpha
J
P25786, Proteasome subunit alpha type−1
K
P20618, Proteasome subunit beta type−1
L
P15976, GATA1
M
P23769, GATA2
N
Q9NY12, GAR1
O
O60832, DKC1
Peptide precursor intensity
Cell type and donor code
CMP_132
CMP_185
CMP_234
CMP_235
CMP_236
GMP_132
GMP_185
GMP_234
GMP_235
GMP_236
HSC_132
HSC_185
HSC_234
HSC_235
HSC_236
MEP_132
MEP_185
MEP_234
MEP_235
MEP_236

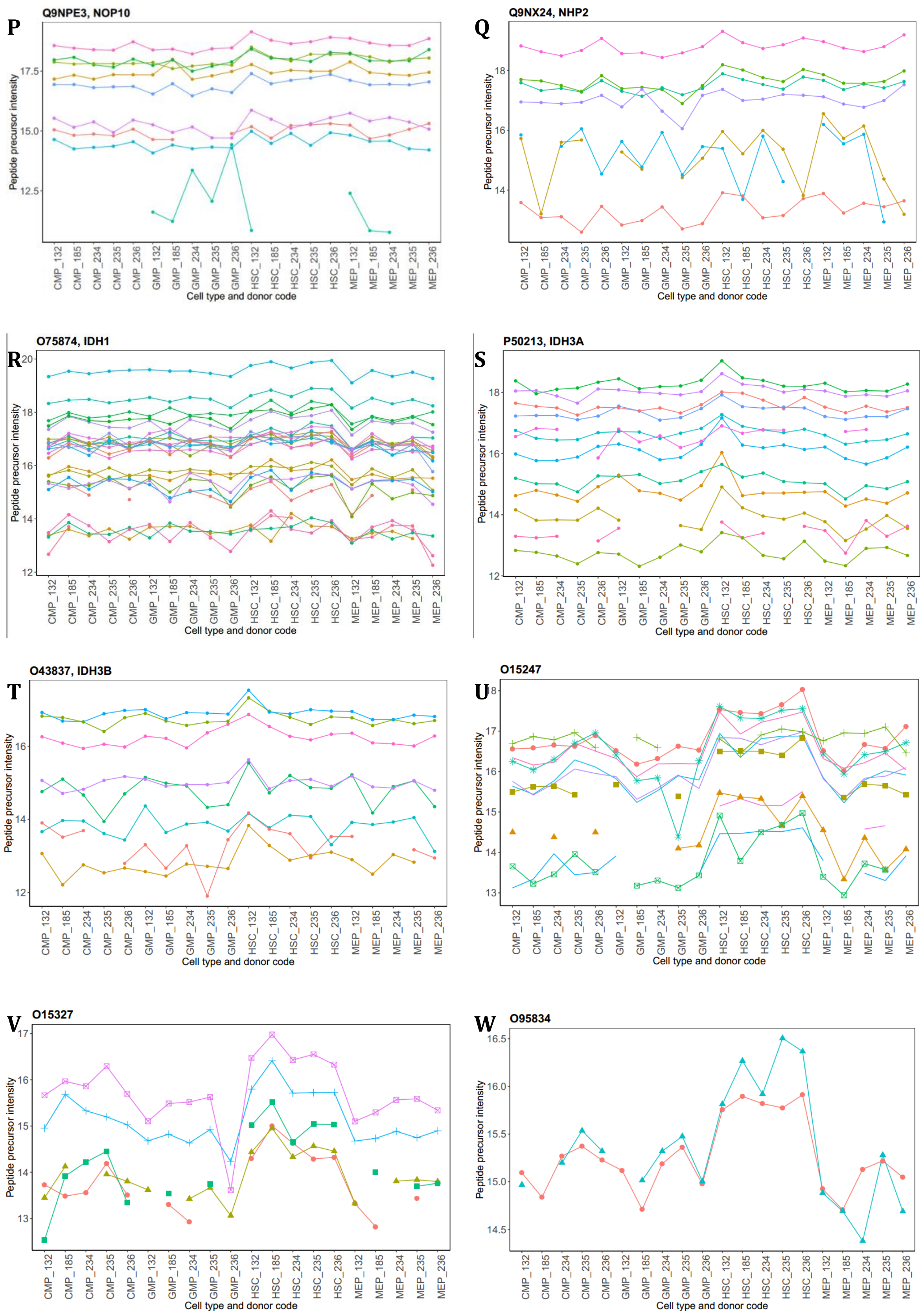
P
Q9NPE3, NOP10
Q
Q9NX24, NHP2
R
O75874, IDH1
S
P50213, IDH3A
T
O43837, IDH3B
U
O15247
V
O15327
W
O95834
Peptide precursor intensity
Cell type and donor code
CMP_132
CMP_185
CMP_234
CMP_235
CMP_236
GMP_132
GMP_185
GMP_234
GMP_235
GMP_236
HSC_132
HSC_185
HSC_234
HSC_235
HSC_236
MEP_132
MEP_185
MEP_234
MEP_235
MEP_236

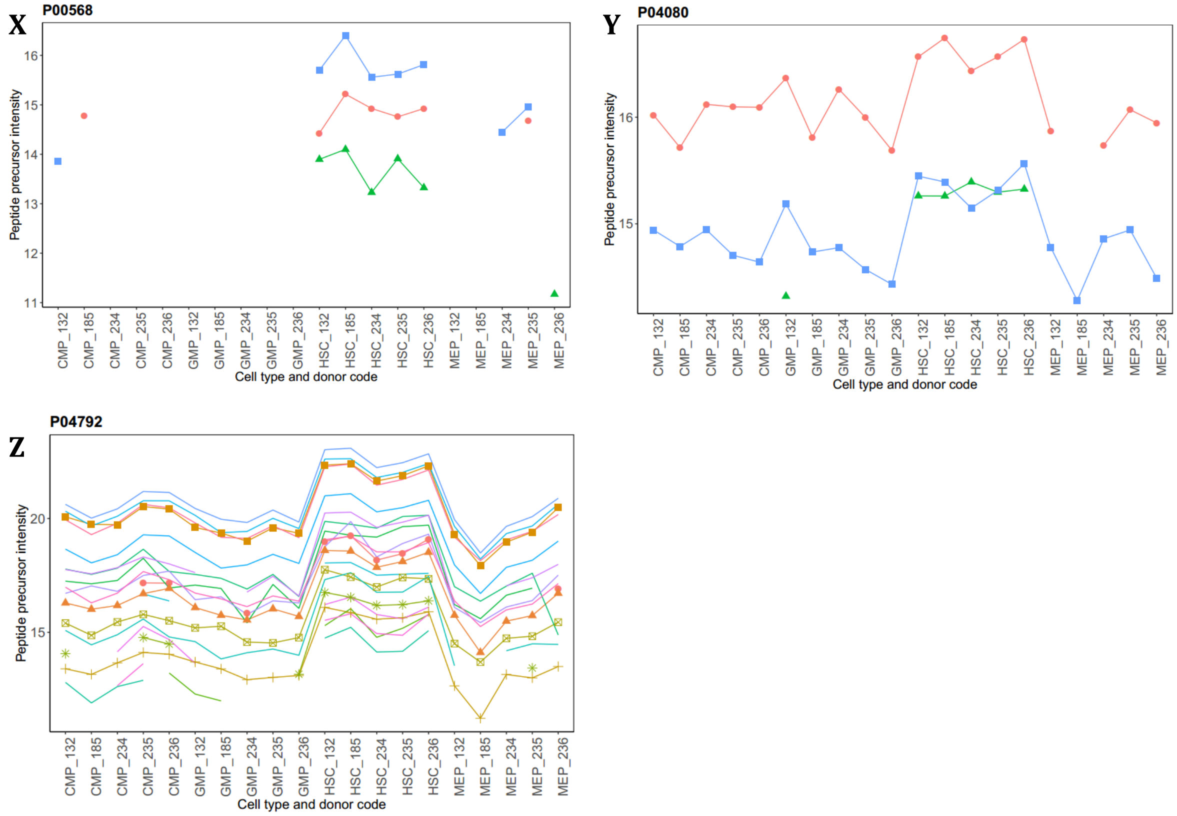

Fig. S15. Peptide precursor intensities for selected proteins. Intensities were calculated by Spectronaut by summing the corresponding fragment ion intensities. Up to ten most abundant peptide precursors (based on averages across samples) are shown with their raw intensities (before normalization). (A)-(G) Examples for proteins with cell type-specific expression patterns, as discussed in the main text; (H)-(K) selected non-regulated proteins involved in main cellular processes; (L)-(M) identified transcription factors from the Hox and Gata gene families; (N)-(T) proteins differentially regulated compared to their mRNAs in the HSC cell type discussed in the manuscript; (U)-(Z) proteins with higher abundance in the HSC cell type.

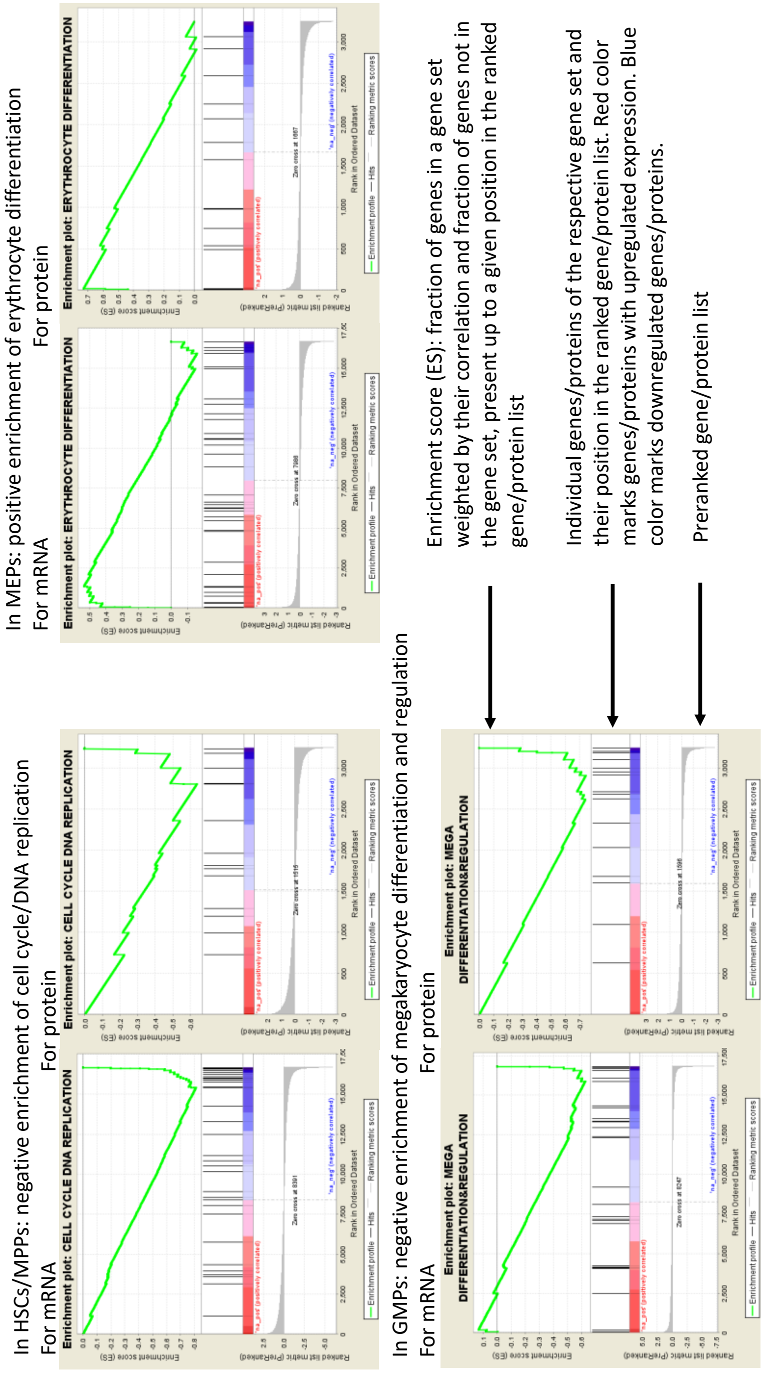

Fig. S16. Gene set enrichment analysis (GSEA) was performed for gene/protein expression in HSCs/MPPs, MEPs, GMPs using CMPs as reference. Similar plots were obtained comparing individual cell types to the average of the three remaining cell types. The respective gene sets are listed in the plot headings. Positive enrichments are marked by preferential occurrence of gene set members in the red area (genes/proteins with upregulated expression). Negative enrichments are marked by preferential occurrence of gene set members in the blue area (genes/proteins with downregulated expression). See Experimental Procedures, (48).

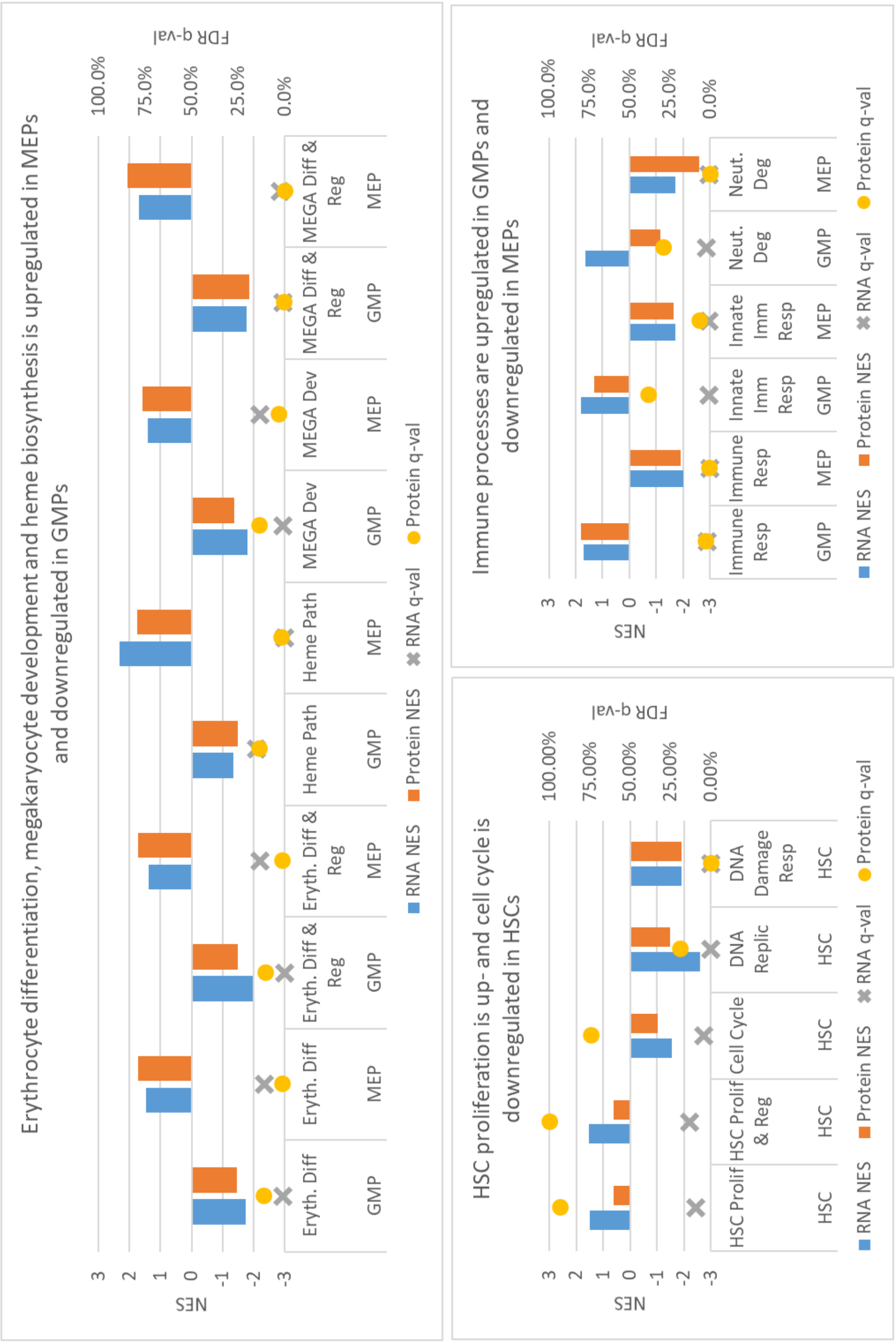

Fig. S17. HSCs/MPPs (referred to as HSCs), GMPs, MEPs showed the expected changes in GO processes on the mRNA and protein level. Shown on the x-axis are the GO processes as outlined in Fig. 4B. Results were aligned for transcriptomics and proteomics with exceptions occurring only with non-significant FDR values of > 0.25. Individual cell types were compared to the average of the three remaining cell subpopulations. Abbreviations: NES - normalized enrichment score, MEGA - megakaryocyte.

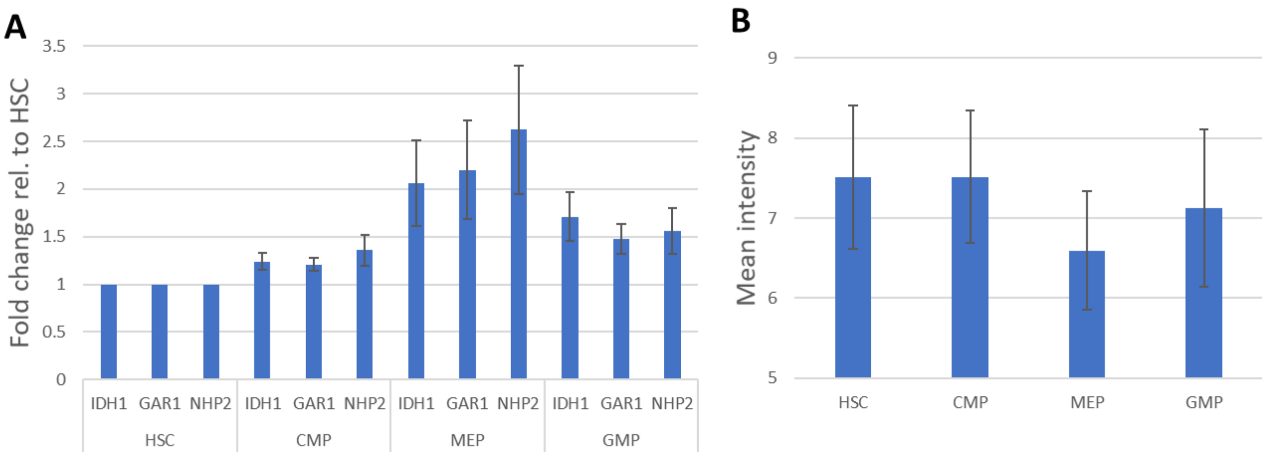

Fig. S18. (A) qPCR validation of mRNA expression in various HSPC subpopulations. Three entities (IDH1, GAR1, NHP2) were selected for validation. ACTB was used as housekeeping control gene. Expression values were calculated using a Delta CT approach. Bars represent standard errors. (B) Flow cytometry validation of protein expression in various HSPC subpopulations. Shown is the mean IDH1 intensity, bars represent coefficient of variation, values are relative to the isotype control. HSC/MPP are referred to as HSC.

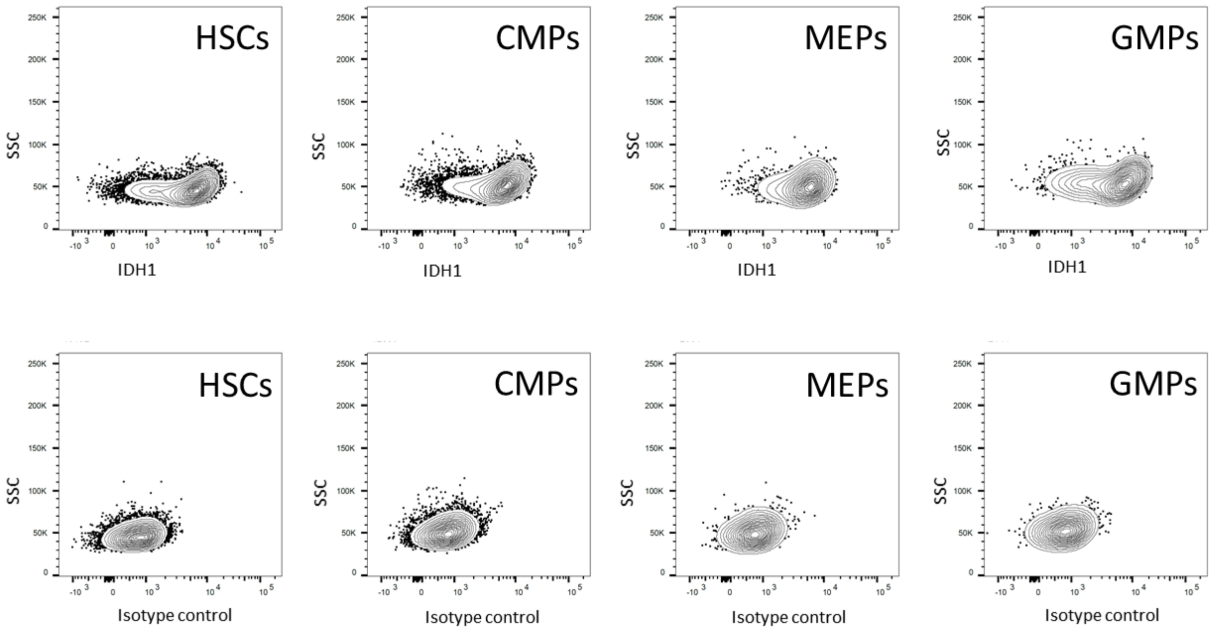

Fig. S19. Representative flow cytometry plots of IDH1/isotype control intensities in different HSPC subpopulations. HSC/MPP is referred to as HSC.

Cell Type	Donor-132	Donor-185	Donor-234	Donor-235	Donor-236
**HSC**	25,000	25,000*	25,000*	25,000*	25,000*
**CMP**	25,000	25,000*	25,000*	25,000*	25,000*
**GMP**	25,000	25,000*	25,000	25,000*	25,000
**MEP**	25,000	25,000*	25,000	25,000*	25,000*
**Age**	28	51	34	29	57
**Sex**	m	m	m	m	f

Table S1. Donor and sample characteristics. Number of FACS-isolated cells that were used for the proteomic analyses; left-over cells from samples are highlighted with an asterisk and were used for library generation via DDA measurement. Donor age is given in years, donor gender is depicted as m=male, f=female. HSC/MPP is referred to as HSC.

Sorted cells x 1,000	ng peptides injected	Mean Ids for triplicates	Cumulative Ids for triplicates	Common Ids for triplicates	Consistency for triplicates	Cumulative Ids for dilution series	Intersection for dilution series	Median CV for triplicates
200	2327	6955	7457	6477	0.869	7457	7457	7.9
100	1164	6763	7343	6208	0.845	7833	6967	9.1
50	582	6264	6868	5702	0.830	7977	6460	13.9
25	291	5751	6381	5150	0.807	8061	5925	9.2
12.5	145	4833	5510	4207	0.764	8102	5135	10.9
6.25	73	2248	2709	1849	0.683	8107	2607	17.4

Table S2. Results from the FACS-isolated CD34+ hematopoietic stem/progenitor cell experiment

Sample Id	Ids per sample
CMP_132	6001
CMP_185	5908
CMP_234	5888
CMP_235	5792
CMP_236	5841
GMP_132	5940
GMP_185	5679
GMP_234	6061
GMP_235	5926
GMP_236	5863
HSC_132	5608
HSC_185	5689
HSC_234	5809
HSC_235	5744
HSC_236	5731
MEP_132	5952
MEP_185	5956
MEP_234	5920
MEP_235	5912
MEP_236	5805

	Protein groups
Cumulative Ids (precursor Q-value < 0.01)	7718
Cumulative Ids (protein Q-value < 0.01)	6043
Common Ids for all samples	4351
**Filtered ≥ 2 precursors with full series of cell type or donor**	**4131**

Table S3. Results from the FACS-isolated hematopoietic stem and progenitor cells experiment

A

ng peptides injected	Mean Ids for triplicates	Cumulative Ids for triplicates	Common Ids for triplicates	Consistency for triplicates	Cumulative Ids for dilution series	Intersection for dilution series	Median CV for triplicates
DIA							
2000	7406	7857	6930	0.882	7857	7857	10.5
1000	7340	7807	6872	0.880	8100	7564	9.4
500	7224	7689	6743	0.877	8199	7364	9.6
250	6865	7352	6383	0.868	8232	7069	9.3
125	6472	6985	5958	0.853	8249	6740	8.9
62.5	5714	6372	5044	0.792	8256	6181	12.5
31.3	4138	4640	3662	0.789	8260	4566	7.8
15.6	3103	3598	2655	0.738	8261	3509	9.0
7.8	1930	2360	1547	0.656	8261	2329	9.3
3.9	1527	1855	1233	0.665	8262	1756	8.7
DDA							
2000	4705	5345	4092	0.766	5345	5345	
1000	4612	5278	4007	0.759	5803	4820	
500	4497	5167	3849	0.745	6018	4544	
250	4325	4912	3751	0.764	6124	4294	
125	4132	4653	3638	0.782	6192	4088	
62.5	3819	4323	3329	0.770	6220	3865	
31.3	3263	3708	2837	0.765	6239	3421	
15.6	2776	3160	2398	0.759	6258	2913	
7.8	2076	2414	1753	0.726	6265	2253	
3.9	1382	1611	1171	0.727	6273	1508	

B

CD34 cells processed	Mean Ids for triplicates	Cumulative Ids for triplicates	Common Ids for triplicates	Consistency for triplicates	Cumulative Ids for dilution series	Intersection for dilution series	Median CV for triplicates
DIA							
200'000	6955	7457	6477	0.869	7457	7457	7.9
100'000	6763	7343	6208	0.845	7833	6967	9.1
50'000	6264	6868	5702	0.830	7977	6460	13.9
25'000	5751	6381	5150	0.807	8061	5925	9.2
12'500	4833	5510	4207	0.764	8102	5135	10.9
6'250	2248	2709	1849	0.683	8107	2607	17.4

Table S4. Summary of the protein identification and peptide quantification **r**esults from the HEK293 peptide dilution series (A) and CD34 FACS-sorted cell numbers (B) experiment
